# Fine Tuning the Pore Surface in Zirconium Metal–Organic Frameworks for Selective Ethane/Ethylene Separation

**DOI:** 10.1021/acsaenm.2c00079

**Published:** 2022-10-14

**Authors:** Yuchen Hu, Yanshu Shi, Yi Xie, Rebecca Shu Hui Khoo, Christian Fiankor, Xu Zhang, Banglin Chen, Jian Zhang

**Affiliations:** † Department of Chemistry, University of Nebraska—Lincoln, Lincoln, Nebraska 68588, United States; ‡ Department of Chemistry, University of Texas at San Antonio, One UTSA Circle, San Antonio, Texas 78249, United States; § The Molecular Foundry, Lawrence Berkeley National Laboratory, Berkeley, California 94720, United States; ∥ School of Chemistry and Chemical Engineering, Jiangsu Engineering Laboratory for Environment Functional Materials, Jiangsu Collaborative Innovation Center of Regional Modern Agriculture & Environmental Protection, Huaiyin Normal University, No.111 West Changjiang Road, Huaian, Jiangsu 223300, China

**Keywords:** gas adsorption, porous materials, metal−organic frameworks, ethane/ethylene separation, adsorption selectivity, zirconium-based MOFs

## Abstract

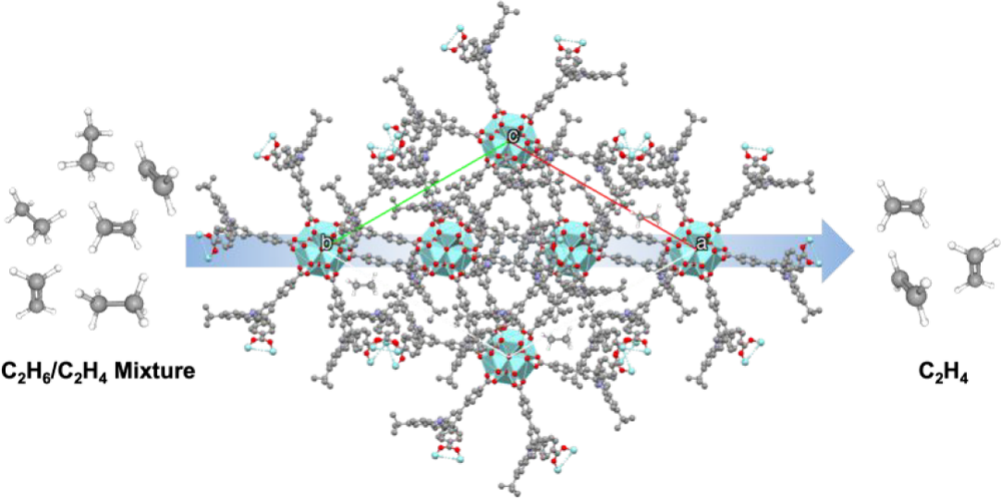

Ethylene is an important chemical feedstock for production of polymers and high-value organic chemicals, and yet its conventional purification process is plagued with high consumption of energy. Metal–organic frameworks (MOFs) provide a suitable adsorption platform for selective ethane/ethylene separation thanks to their structural diversity, tunable pore characteristics, designable pore sizes, and high pore volumes. Although there are empirical design rules like avoiding open metal sites and creating nonpolar pore surfaces for development of adsorptive MOFs, it is still challenging to design robust MOFs that can realize direct ethane-selective separation. Herein, we systematically designed and synthesized three Zr-MOFs based on the assembly of angular ligands and 12-connected Zr_6_ clusters that feature the **pcu** network structure. By changing the size and flexibility of the substituent on the angular ligand, we were able to prevent interpenetration and identified NPF-802, which exhibits good C_2_H_6_/C_2_H_4_ separation performance that is attributed to the bulky and inert *tert*-butyl groups of its carbazole ligand. This work provides insights for design of ligands of MOFs with suitable pore environments to address important and challenging gas separations.

## Introduction

With a global production capacity of exceeding 170 million tonnes per year,^1^ ethylene (C_2_H_4_) is one of the most important chemical feedstocks in petrochemical and materials industries for producing polymers and high-value organic chemicals.^2,3^ The primary industrial production of C_2_H_4_ is steam cracking (or thermal decomposition) of ethane (C_2_H_6_) and liquidized petroleum gases.^4^ The removal of the impurities (mostly C_2_H_6_) from C_2_H_4_/C_2_H_6_ mixtures is usually accomplished by heat-driven cryogenic distillation under harsh conditions (typically at 5–28 bar and 180–258 K),^5,6^ which is extremely energy intensive. The total energy used for such separation is estimated to be about 7.3 GJ per tonne of C_2_H_4_.^7^ For context, 1 GJ of energy is equal to about 8 gallons of gasoline. To avoid such a high consumption of energy, it is urgent to explore alternative technologies and materials that can efficiently separate and purify C_2_H_4_ under mild conditions with lower energy consumption, and adsorptive-based gas separation is such a good alternative technique.^8,9^

Metal–organic frameworks (MOFs) with structural diversity, tunable pore characteristics, designable pore sizes, and high pore volumes have emerged as promising solid adsorbents to address challenging gas separations,^10−18^ like separation of olefins from paraffins.^19−25^ Incorporation of functional sites into MOFs can enhance binding interactions for polar hydrocarbons, while precise control over the pore sizes can exclude larger molecules, both resulting in improved separation performance. However, the separation of C_2_H_4_ and C_2_H_6_ is challenging due to their similar physical properties and molecular dimensions (3.28 × 4.18 × 4.84 Å^3^ for C_2_H_4_ and 3.81 × 4.08 × 4.82 Å^3^ for C_2_H_6_).^26^ Compared to C_2_H_4_-selective MOFs,^27,28^ C_2_H_6_-selective MOFs^29,30^ are highly desirable, particularly in the cases where minor C_2_H_6_ impurities need to be removed from C_2_H_4_, as they can produce high-purity alkenes directly during the adsorption, simplifying the process and resulting in an increase in productivity.^31^ It is estimated that C_2_H_6_-selective MOFs are more economical for C_2_H_4_/C_2_H_6_ separation and offer ∼40% energy savings when compared with C_2_H_4_-favored MOFs.^32,33^

The key point for designing C_2_H_6_-selective MOFs is to achieve the combination of good selectivity and high uptake capacity, which however remains a daunting challenge. Since C_2_H_6_ has a higher polarizability than C_2_H_4_ (44.7 × 10^–25^ vs 42.52 × 10^–25^ cm^3^), dispersion and induction interactions such as C–H···π interactions between C_2_H_6_ and MOFs would make major contributions to C_2_H_6_-selective adsorbents.^34^ MOF materials with a pore structure enriched with nonpolar and inert surfaces (e.g., featuring aromatic or aliphatic moieties) may favor the preferential adsorption of C_2_H_6_ over C_2_H_4_.^35−37^ To date, C_2_H_6_-selective MOFs are still scarce^38−44^ due to the difficulty of discriminating C_2_H_6_ over C_2_H_4_ or low C_2_H_6_ uptake resulting from small pore volume.^45,46^ Only a handful of them show favorable adsorption capacity for C_2_H_6_ (>3 mmol/g at 1 bar and 298 K).^47−50^ Nevertheless, a couple of design rules seem to hold for the design of MOF platforms for C_2_H_6_-selective separation. First, open metal sites are undesirable as they preferentially adsorb water molecules and unsaturated hydrocarbons such as C_2_H_4_, thereby dampening the adsorption capacity and selectivity for C_2_H_6_.^51^ Second, a MOF with a pore structure enriched with nonpolar surfaces such as aromatic or aliphatic moieties and pore dimensions that match the size of C_2_H_6_ may favor its preferential adsorption. Introducing and modifying ligand functional groups can be a feasible approach to regulate MOF pore environments to endow them with specific binding affinities for C_2_H_6_.^52−55^

The highly robust 12-connected Zr-MOF is an ideal design platform for C_2_H_6_-selective MOFs due to the absence of open metal sites. Typically, a linear ditopic ligand is used to generate UiO-type 12-connected Zr-MOFs.^56^ Recently, the Li group has adopted angular ditopic ligands to construct 12-connected Zr-MOFs, namely, BUT-66 and BUT-67.^57^ From the topological point of view, if each pair of angular ligands are considered as an edge and each 12-connected Zr_6_(μ_3_-O)_4_(μ_3_-OH)_4_ (Zr_6_) cluster is regarded as a corner, a **pcu** net would best represent the interconnection in these MOFs. It is noted that the **pcu** net in BUT-66 and BUT-67 is highly open and results in 2-fold interpenetration. Therefore, we reasoned that if the 5-position of the central phenyl ring of the angular ligand were functionalized with a nonpolar or inert group to boost C_2_H_6_-binding affinities, the resulting Zr-MOFs could offer an ideal platform for the design of C_2_H_6_-selective MOFs. In this work, we systematically introduced three aromatic groups, namely, *N*,*N*-diphenylamine, 9-carbazole, and 3,6-di-*tert*-butyl-9-carbazole, to fine tune the pore surface of the resulting Zr-MOFs, namely, NPF-800, NPF-801, and NPF-802, respectively (NPF = Nebraska Porous Frameworks). We show that all three bulky aromatic groups effectively eliminate the formation of the interpenetrated structure as observed in BUT-66; and more interestingly, the introduction of a *tert*-butyl group in NPF-802 not only retains the permanent framework porosity but also enables excellent preferential adsorption of C_2_H_6_ over C_2_H_4_. This work points to a new direction for functionalizing pore structures for gas adsorption and separation using angular ligands.

## Results and Discussion

### Synthesis and Crystal Structures of NPF-800 Series

**Scheme 1:**
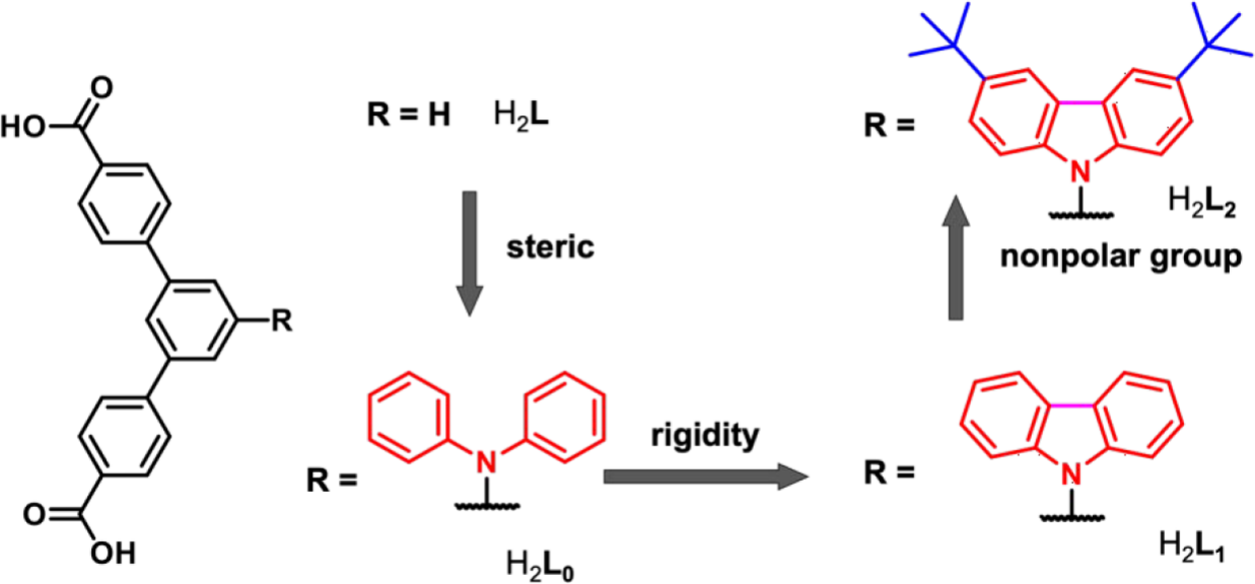
Systematic Tuning of the Steric Effect and Polarity of Angular Ligands

**Figure 1:**
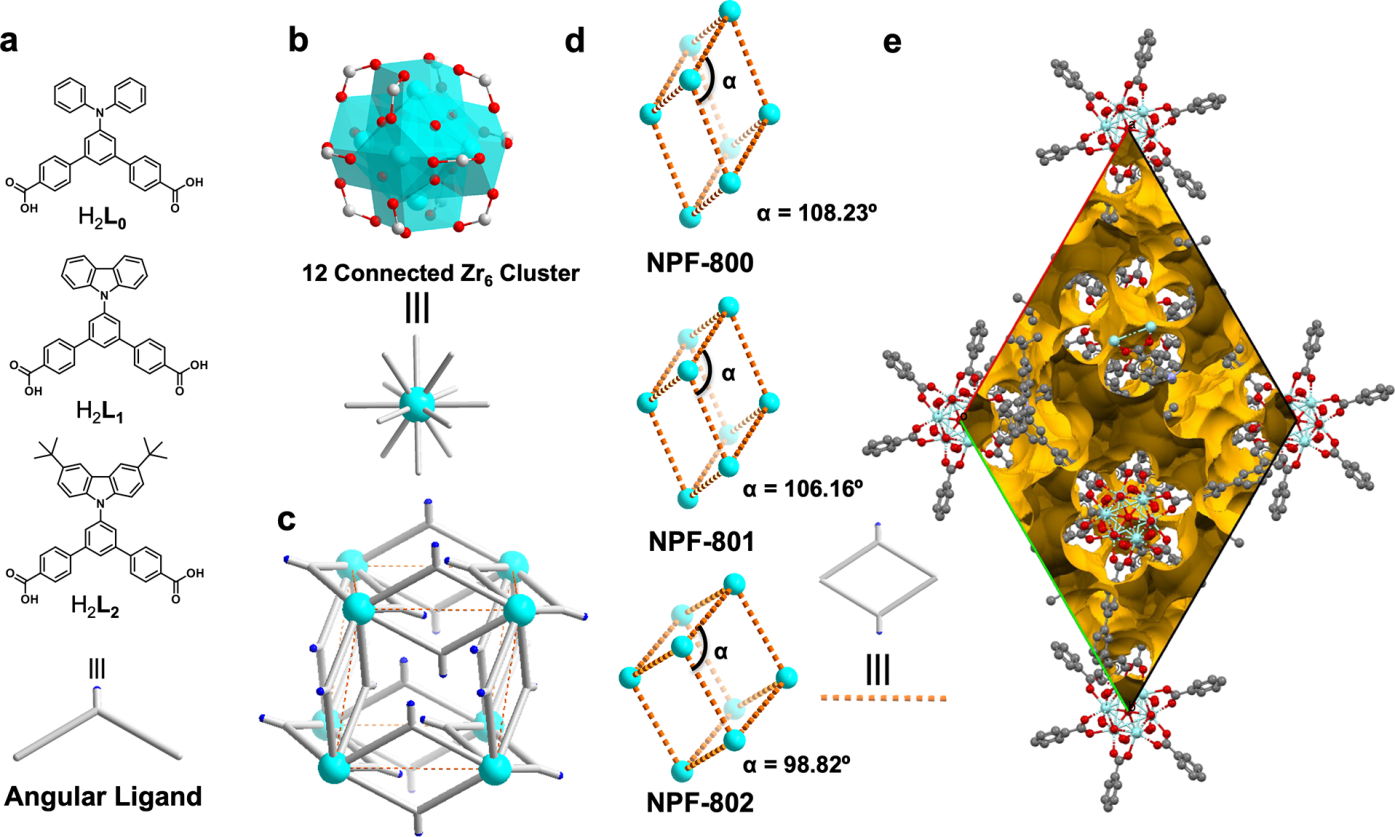
(a) Ditopic angular ligands and (b) 12-coordinated Zr_6_ cluster and their simplified representations. (c) Simplified representation of the 3D **pcu** network. (d) Simple pseudo-cubic lattice in NPF-800, NPF-801, and NPF-802, viewed from the pseudo-(110) direction. (e) Internal pore structure of NPF-802 (probe of diameter 1.0 Å).

The structure prototype of the angular *meta*-terphenyl dicarboxylic acid (H_2_**L**) was used as the ligand for the synthesis of BUT-66, which is an interpenetrated Zr-based MOF.^57^ Here, we first introduce a bulky *N*,*N*-diphenylamino group to the 5-position of the central phenyl ring via a Cu-catalyzed Ullmann coupling reaction to afford the functionalized angular ligand H_2_**L**_**0**_ (Scheme 1). Single crystals of NPF-800 were obtained from solvothermal reactions of ZrCl_4_·8H_2_O and H_2_**L**_**0**_ in *N*,*N*-dimethylformamide (DMF) at 120 °C for 48 h (see Supporting Information for synthetic details). NPF-800 crystallizes in the *R*3̅ space group of the trigonal system and has a 3-D framework consisting of Zr_6_ clusters and angular ligand **L**_**0**_^2–^ (Figure 1, Table S1). Like BUT-66, each 12-connected Zr_6_ cluster in NPF-800 coordinates with six pairs of angular ligands (**L**_**0**_^2–^), and each pair of ligands bridges two Zr_6_ clusters, and the simplified topology can be described as a distorted **pcu** network formed by the interconnection of these two types of building units (Figure 1). To our delight, the introduction of the bulky diphenylamino group to the angular ligand indeed avoided interpenetration.^57^ Unfortunately, the yield of highly crystalline NPF-800 is low and attempts to increase the product yield adversely resulted in powders with poor crystallinity (Figure S6), likely due to the framework flexibility contributed by the free rotation of the diphenylamino group of angular ligand **L**_**0**_^2–^. Thus, further modification of the angular ligand was carried out to achieve more rigid frameworks with permanent porosity.

It was hypothesized that reducing the rotational freedom of the diphenylamino group by locking the two phenyl groups as a carbazole could improve the crystallization. In addition, the 3,6-positions of carbazole offer an excellent opportunity to incorporate more nonpolar or inert groups such as *tert*-butyl for better binding affinities for C_2_H_6_. Thus, two new angular ligands, H_2_**L**_**1**_ and H_2_**L**_**2**_ (Scheme 1), that are functionalized with 9-carbazole and 3,6-di-*tert*-butyl-9-carbazole, respectively, were synthesized via base promoted *N*-arylation reactions and further employed to construct Zr-MOFs (see Supporting Information for details). NPF-801 and NPF-802 were obtained from the solvothermal reactions of ZrCl_4_·8H_2_O and H_2_**L**_**1**_ and H_2_**L**_**2**_, respectively (see Supporting Information for synthetic details). With careful adjustment of the amount of modulating agents (benzoic acid, trifluoroacetic acid, and acetic acid) in the reaction systems, single crystals suitable for X-ray diffraction structural analysis were obtained.

Overall, NPF-801 and NPF-802 are isostructural to NPF-800 and crystallize in the trigonal system of *R*3̅ space group (Table S1). Like NPF-800, the simplified topology of both NPF-801 and NPF-802 belongs to the **pcu** net with no interpenetration (Figure 1). However, setting aside the similar structure and topology, it is interesting to compare the change of cell parameters of the NPF-800 series. Specifically, NPF-800 has the longest *c* axis (32.81 Å) but the shortest *a*/*b* axes (21.47 Å) and thus the smallest cell volume of 20 013.2 Å^3^. On the other hand, NPF-802 has the shortest *c* axis (28.26 Å), the longest *a*/*b* axes (29.76 Å), and the largest cell volume of 21 680.3 Å^3^ (Table S1). NPF-802 also has the smallest void fraction of 21.5%, compared with 25.9% and 27.8% for NPF-800 and NPF-801, respectively. Overall, the introduction of the rigid, bulky, and nonpolar 3,6-di-*tert*-butyl-9-carbazole leads to a less deformed **pcu** network, indicated by the smaller pseudo-lattice-angle of 98.82° compared with 106.16° and 108.23° for NPF-801 and NPF-800, respectively.

**Figure 2:**
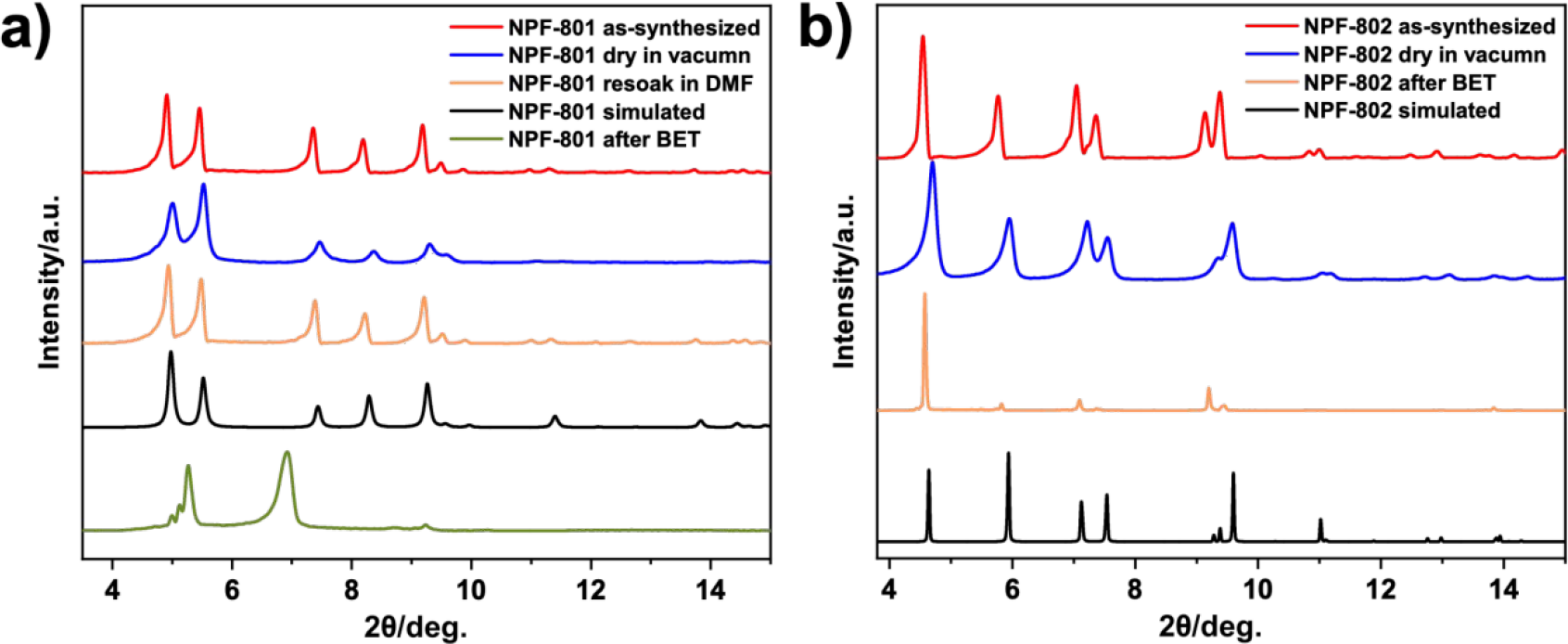
PXRD patterns of (a) NPF-801 and (b) NPF-802.

The powder X-ray diffraction (PXRD) patterns of the solvated and desolvated NPF-801 and NPF-802 exhibit excellent agreement with the simulation, confirming the bulk purity of the material (Figure 2). Both MOFs exhibit excellent thermal stability, with a high decomposition temperature around 450–500 °C based thermogravimetric analysis (Figures S12 and S13). Excellent crystallinity was well retained after treatment in H_2_O and basic (pH = 11), and acidic (pH = 1) conditions (Figure S14). Such characteristics suggest that both materials are good candidates for gas adsorption applications.

### Porosity and Gas Separation Performance of NPF-800 Series

**Figure 3:**
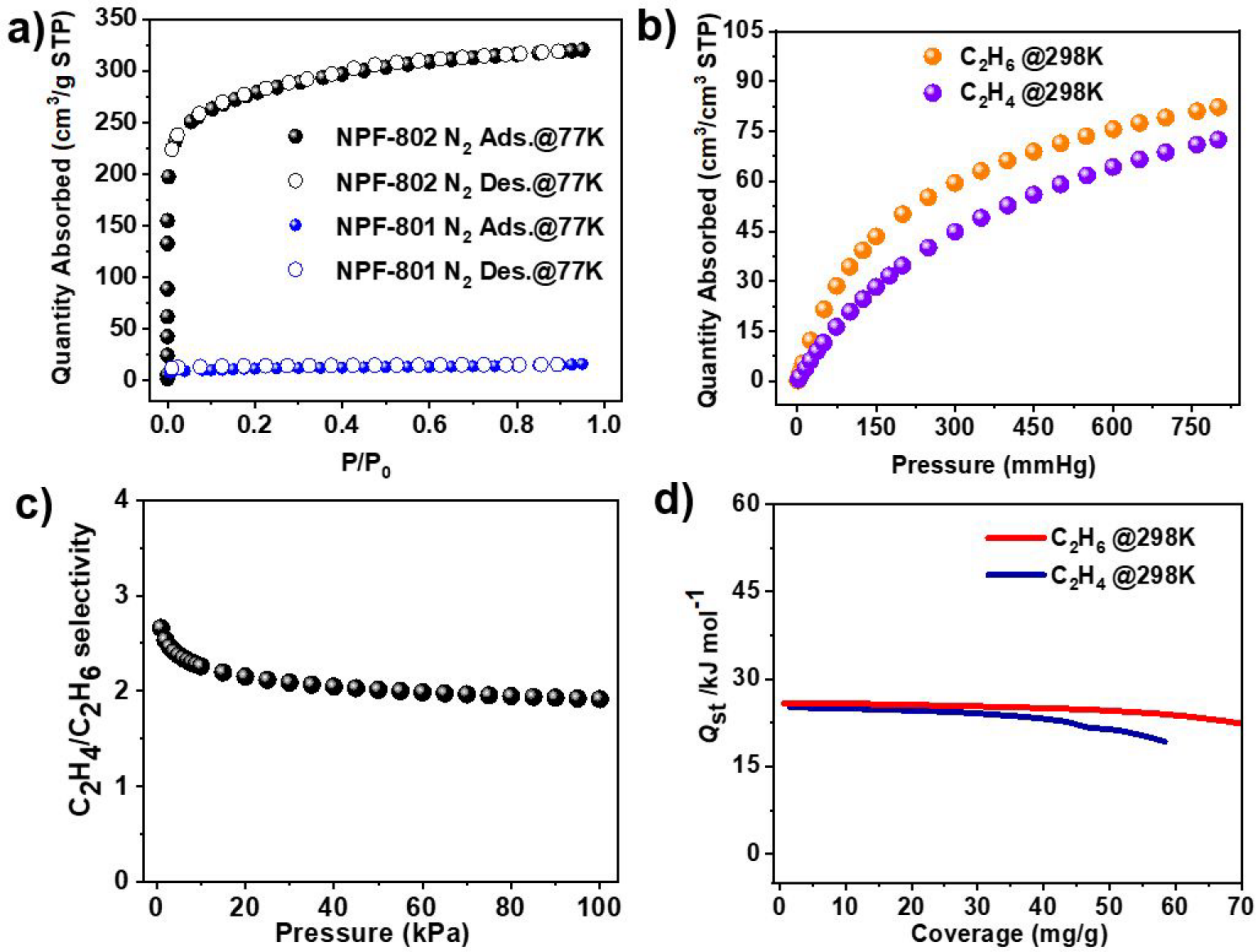
(a) N_2_ sorption isotherms for NPF-802 and NPF-801 at 77 K; (b) C_2_H_4_ and C_2_H_6_ sorption isotherms for NPF-802 at 298 K; (c) IAST selectivity of NPF-802 for an equimolar C_2_H_4_/C_2_H_6_ mixture at 298 K; (d) Coverage-dependent adsorption enthalpy of C_2_H_4_ and C_2_H_6_ calculated by the virial fitting method.

The permanent porosity of NPF-802 was established by nitrogen (N_2_) gas sorption experiments at 77 K. As shown in Figure 3a, NPF-802 can adsorb a large amount of N_2_ (311.9 cm^3^ g^–1^) at 77 K and *P*/*P*_0_ = 0.95. The experimental Brunauer–Emmett–Teller (BET) surface area (SA_BET_) and pore volume were calculated to be moderate at 1080 m^2^ g^–1^ and 0.524 cm^3^ g^–1^, close to the theoretical value of 1100 m^2^ g^–1^ and 0.48 cm^3^ g^–1^, respectively. The slight difference in pore volume, in part, can be attributed to the structural change at different temperatures: experimental pore volume is based on the BET measurement at 77 K, while the theoretical pore volume is based on the single crystal structure determined at 100 K. The pore size distribution calculated by density functional theory (DFT) was 8.2 Å (Figure S15). NPF-802 also displayed good adsorption capability for CO_2_ (2.2 mmol g^–1^) at 298 K and 1 bar (Figure S16). Different from NPF-802, NPF-801 does not have significant adsorption of N_2_ at 77 K (16.2 cm^3^ g^–1^) (Figure 3a). Although its CO_2_ adsorption amount of 3.6 mmol g^–1^ at 195 K is likely due to its smaller kinetic diameter (Figure S17),^58^ it is still much lower than the theoretical adsorption value (15.6 mmol g^–1^). Indeed, PXRD patterns after the N_2_ adsorption experiment indicate that NPF-801 undergoes a phase change after BET measurement (Figure 2a). Therefore, NPF-801 could not maintain the framework integrity after activation due to its irreversible structural flexibility, and thus it is not suitable for gas adsorption and separation. In contrast, the presence of bulky *tert*-butyl groups enhances the structural robustness of NPF-802, which remains highly crystalline and porous after BET measurement (Figure 2b) and was thus chosen for further gas separation studies.

We first examined the single-component adsorption isotherms of NPF-802 for C_2_H_6_ and C_2_H_4_ at 298 K up to 1 bar. As shown in Figure 3b, NPF-802 exhibits obvious preferential adsorption of C_2_H_6_ over C_2_H_4_. The amount of uptake of C_2_H_6_ (3.67 mmol g^–1^) is higher than that of C_2_H_4_ (3.24 mmol g^–1^) at 1 bar and 298 K with NPF-802, affording a C_2_H_6_/C_2_H_4_ uptake ratio of 113%. Ideal adsorbed solution theory (IAST) was used to calculate the adsorption selectivity of NPF-800 for C_2_H_6_/C_2_H_4_ mixtures at 298 K. As shown in Figure 3c, NPF-802 exhibits a high C_2_H_6_/C_2_H_4_ selectivity up to 1.9 at 1 bar. Overall, these results indicate that we successfully realized “reversed C_2_H_6_/C_2_H_4_ adsorption” in NPF-802. This interesting reverse C_2_H_6_/C_2_H_4_ selectivity is likely due to the two *tert*-butyl groups on the carbazole and the inert pore surface. Indeed, isosteric enthalpies of adsorption (*Q*_st_) for NPF-802 were calculated by virial equation based on the isotherms collected at 273 and 298 K (Figures S18 and S19), and the initial *Q*_st_ value of C_2_H_6_ for NPF-802 is 25.9 kJ/mol, slightly higher than that of C_2_H_4_ (25.2 kJ/mol). This result is consistent with the stronger thermodynamic affinity of NPF-802 toward C_2_H_6_ than C_2_H_4_.

**Figure 4:**
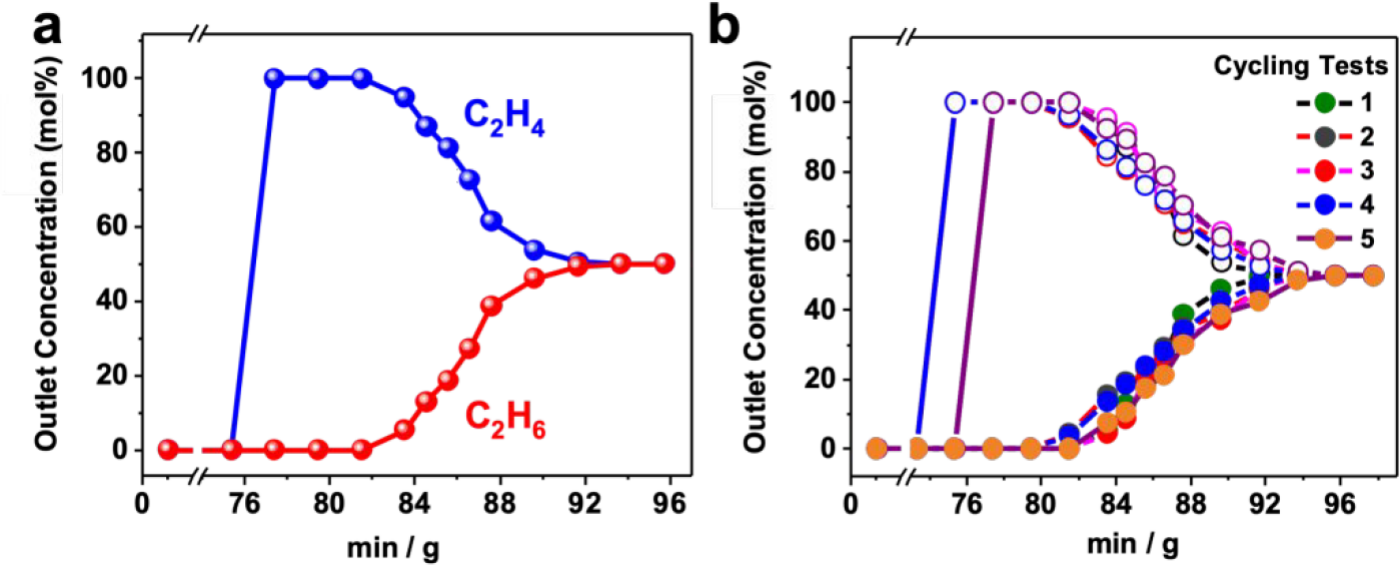
(a) Curves of breakthrough and (b) cycling tests for a mixture of C_2_H_6_/C_2_H_4_ (50/50) in a column packed with NPF-802 at 298 K and 1 bar.

Next, the C_2_H_6_/C_2_H_4_ separation performance was further evaluated by experimental breakthrough studies in which an C_2_H_6_/C_2_H_4_ (50/50) mixture was flowed over a packed column of NPF-802 at a rate of 1 mL min^–1^ at 298 K (see Supporting Information for details). Indeed, complete separation of C_2_H_6_ from the C_2_H_6_/C_2_H_4_ mixture can be achieved under ambient conditions (Figure 4a). Furthermore, the separation performance of NPF-802 under ambient conditions was studied by multiple mixed-gas (C_2_H_6_/C_2_H_4_ at 50/50) column breakthrough tests, which show similar breakthrough times for both C_2_H_6_ and C_2_H_4_ within five continuous cycles, confirming the good recyclability for practical C_2_H_6_/C_2_H_4_ separation (Figure 4b).

## Conclusion

In summary, we have synthesized three novel Zr-MOFs featuring **pcu** topology network structures built from the assembly of angular ligands and 12-connected Zr_6_ clusters. By introducing bulky groups (diphenyl amino, carbazole, and *tert*-butyl carbazole groups) to the angular ligands, we avoid the commonly observed interpenetration of the **pcu** net. One of these three isostructural MOFs, NPF-802, showed robust permanent porosity and exhibited preferential adsorption of C_2_H_6_ over C_2_H_4_, affording the unusual reversed C_2_H_6_/C_2_H_4_ separation. In comparison to NPF-801, it is likely that the bulky and inert *tert*-butyl groups of carbazole in NPF-802 create suitable pore structure for preferential interactions with C_2_H_6_ over C_2_H_4_. This work not only reports good C_2_H_6_/C_2_H_4_ separation performance but also provides some guidance to design novel ligands of MOFs with suitable pore environments to address important and challenging gas separations.

## Supporting Information Available

Synthesis, experimental data and procedures, X-ray diffraction, NMR spectra of digested MOFs, stability, N_2_ adsorption isotherms (PDF)

## Accession Codes

CCDC 2191183–2191185 contain the supplementary crystallographic data for this paper. These data can be obtained free of charge via www.ccdc.cam.ac.uk/data_request/cif, by emailing data_request@ccdc.cam.ac.uk, or by contacting The Cambridge Crystallographic Data Centre, 12 Union Road, Cambridge CB2 1EZ, UK; fax: + 44 1223 336033.
