# Supplementary material for: Fine Tuning the Pore Surface in Zirconium Metal–Organic Frameworks for Selective Ethane/Ethylene Separation
Source: ACS Appl Eng Mater. 2022 Oct 14;1(1):334–40. doi: 10.1021/acsaenm.2c00079 (PMC9903366; doi:10.1021/acsaenm.2c00079)
Supplement: Supplementary file 1 — em2c00079_si_001.pdf [file em2c00079_si_001.pdf]

Supporting Information

Fine Tuning the Pore Surface in Zirconium Metal-Organic Frameworks for Selective Ethane/Ethylene Separation

Yuchen Hu,[1,†] Yanshu Shi,[2,†] Yi Xie,[2] Rebecca Shu Hui Khoo,[3] Christian Fiankor,[1] Xu Zhang,[4] Banglin Chen,[2,] and Jian Zhang[1,3,*]*

[1]Department of Chemistry, University of Nebraska-Lincoln, Lincoln, Nebraska 68588 United States

[2]Department of Chemistry, University of Texas at San Antonio, One UTSA Circle, San Antonio, Texas 78249 United States

[3]The Molecular Foundry, Lawrence Berkeley National Laboratory, Berkeley, California 94720 United States

[4]School of Chemistry and Chemical Engineering, Huaiyin Normal University, Jiangsu Engineering Laboratory for Environment Functional Materials, Jiangsu Collaborative Innovation Center of Regional Modern Agriculture & Environmental Protection, No.111 West Changjiang Road, Huaian, Jiangsu 223300 China

*Email: jianzhang@lbl.gov; banglin.chen@utsa.edu

S-1 Materials and General Procedures

All solvents and reagents were purchased from commercial suppliers and, unless otherwise noted, used without further purification. Solution ^{1}H and ^{13}C nuclear magnetic resonance NMR measurements were performed on a Bruker FT-NMR spectrometer (400 MHz) or a Bruker FT-NMR spectrometer (300 MHz). Powder X-ray diffraction (PXRD) patterns were taken with a PANalytical Empyrean diffractometer with a PIXcel 3D detector. The copper target X-ray tube was set to 45 kV and 40 mA. Fourier transform infrared spectroscopy (FT-IR) was measured on a Nicolet iS50 FT-IR spectrometer. Gas adsorption isotherms were collected using the surface area analyzer ASAP-2020. N_2 gas adsorption isotherms were measured at 77 K using a liquid N_2 bath. The obtained adsorption-desorption isotherms were evaluated to give the pore parameters, including Brunauer-Emmett-Teller (BET) specific surface area, pore size, and pore volume.

Activation method of the MOF samples and for N_2 gas adsorption measurement: as-synthesized MOF samples were exchanged with fresh DMF at least three times. Then the MOFs samples with 1 mL DMF were added to 20 μL 8 M HNO_3 and heated in an oven at 80 °C for 12 h to remove the unreacted ligand and cluster and modulators. The activated samples were exchanged with fresh DMF three times again and subsequently exchanged with anhydrous ethanol 3 times in 36 h to remove DMF completely. The ethanol exchanged samples were activated with the Samdri®-PVT-3D supercritical CO_2 dryer and immediately used for gas adsorption measurement.

C_2H_2 (99.99%), C_2H_4 (99.99%), C_2H_6 (99.99%), He (99.999%) and mixed gases of C_2H_4/C_2H_6 = 50/50 (v/v) were purchased from Airgas. IAST calculations of adsorption selectivity: the selectivity of preferential adsorption of component 1 (C_2H_6) over component 2 (C_2H_4) can be defined as

$$S_{ads} = \frac{q1/q2}{p1/p2}$$

Where $q1$ and $q2$ are the absolute component loadings of the adsorbed phase in the mixture, and $p1$ and $p2$ are the components' partial pressures. The component loadings and adsorption selectivity S_{ads} for 50/50 C_2H_6(1)/C_2H_4(2) in NPF-800 at 298 K were determined using IAST.

Isosteric heat of adsorption: The binding energy of C_2H_4 and C_2H_6 is reflected in the isosteric heat of adsorption, Q_{st}. The virial equation was employed to calculate the enthalpies of C_2H_4 and C_2H_6 adsorption, where P is pressure (mmHg), N is the adsorbed quantity (mmol g^{-1}), T is the temperature (K), a_i and b_i are virial coefficients, R is the universal gas constant (8.314 J K^{-1} mol^{-1}), and m and n determine the number of coefficients required to adequately describe the isotherm.

$$Q_{st} = -R\sum_{i=0}^{m} a_i N^i$$

$$InP = InN + \frac{1}{T}\sum_{i=0}^{m} a_i N^i + \sum_{i=0}^{n} b_i N^i$$

Isotherm (Q_{st}) of **NPF-802** for C_2H_4 and C_2H_6 reported here are estimated using pure-component isotherms collected at 273 and 298 K.

Breakthrough separation experiments: The breakthrough experiments were conducted in a dynamic gas breakthrough set-up. A stainless-steel column with inner dimensions of 4 mm and a length of 81 mm was used for sample packing. The activated sample (0.489 g **NPF-802**) was then packed into the column. The flow and pressure of binary gas (C_2H_6/C_2H_4 at 50/50, v/v) were controlled by using a pressure control valve and a mass flow controller. The outlet effluent from the column was continuously monitored by gas chromatography (GC-2014, Shimadzu) with a thermal conductivity detector. The column packed with activated sample was first purged with helium gas flow for 1 h at room temperature. The gas mixtures flow rate is 1 mL/min at 1 bar for

(C_2H_6/C_2H_4 at 50/50, v/v) during the breakthrough process. After the breakthrough experiment, the sample was regenerated with helium gas flow (60 mL/min) for about 40 min at 298 K.

S-2 Synthesis and General Characterization

Angular ditopic ligand H_2L_0 was synthesized *via* two coupling reactions followed by saponification in a basic aqueous solution (Scheme S1). Below is the detailed synthesis:

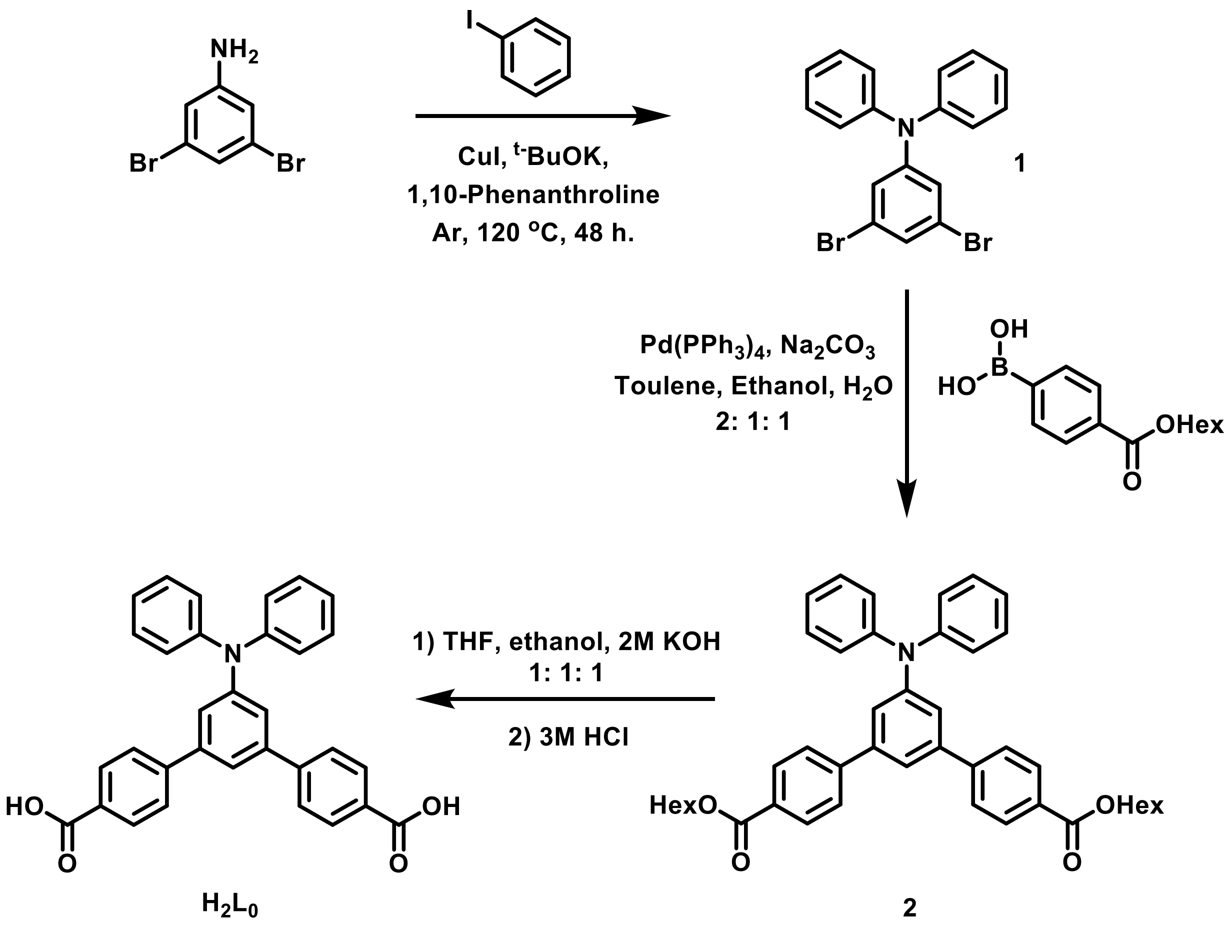

Scheme S1. Synthesis of ligand H_2L_0.

Synthesis of 3,5-dibromo-N,N-diphenylaniline (1): In a 250-mL three-necked flask with ^{t}BuOK (15 mmol), 1,10-Phenanthroline (0.5 mmol), CuI (0.5 mmol) under argon at room temperature. 3,5-dibromoaniline (10 mmol) and iodobenzene (40 mmol) were added. The dried toluene (50 mL) was added via syringe. The reaction mixture was then stirred and heated at 120 ^{0}C for 48 hours. The reaction mixture was then cooled, mixed with 100 mL of diethyl ether, and filtered to remove any insoluble residues. The solvent was removed under reduced pressure and

the residue was then purified by column chromatography on silica gel to obtain the white solid product (compound 5). ^{1}H NMR (400 MHz, Chloroform-*d*) δ 7.33 (dd, *J* = 8.4, 7.3 Hz, 4H), 7.21 (t, *J* = 1.7 Hz, 1H), 7.16 – 7.09 (m, 6H), 7.07 (d, *J* = 1.7 Hz, 2H).

Synthesis of dihexyl 5'-(diphenylamino)-[1,1':3',1''-terphenyl]-4,4''-dicarboxylate (2): Compound 1 (4.03 g, 10 mmol), (4-((hexanoyloxy)carbonyl) phenyl) boronic acid (6.6 g, 25 mmol), Na_2CO_3 (8.5 g, 80 mmol) were dissolved in mixed solvent of toluene-ethanol-water (40 mL: 20 mL: 20 mL). After degassing by argon for 1 h, Pd $(PPh_3)_4$ (0.8 g, 0.68 mmol) was added to the solution. The solution was stirred under argon atmosphere for 48 h under reflux at 100 °C. After filtration, the solvent was removed under reduced pressure, the resulting residue was purified using column chromatography of silica gel to obtain compound 2 in 66% yield. ^{1}H NMR (400 MHz, Chloroform-*d*) δ 8.14 (d, *J* = 8.4 Hz, 4H), 7.66 (d, *J* = 8.4 Hz, 4H), 7.54 (s, 1H), 7.42 (d, *J* = 1.6 Hz, 2H), 7.34 (dd, *J* = 8.5, 7.2 Hz, 4H), 7.28 – 7.22 (m, 4H), 7.11 (td, *J* = 7.3, 1.2 Hz, 2H), 4.39 (t, *J* = 6.7 Hz, 4H), 1.86 – 1.79 (m, 4H), 1.56 – 1.49 (m, 4H), 1.42 (dt, *J* = 7.5, 3.9 Hz, 8H), 0.99 – 0.96 (m, 6H).

Synthesis of 5'-(diphenylamino)-[1,1':3',1''-terphenyl]-4,4''-dicarboxylic acid (H_2L_0): Compound 2 (6.8 g, 10 mmol) was dissolved in a mixture of methanol (60 mL), THF (60 mL) and 2 M KOH (60 mL). The solution was degassed for 1 hour, and then refluxed under argon for 24 h and the solution became clear. The solvent was removed under reduced pressure, and the remaining solid was dissolved in water. 3 M HCl was added to the solution until pH = 2.0. After filtration, the precipitate was recrystallized from DMF/water to obtain a light-yellow solid with a 91 % yield. ^{1}H NMR (400 MHz, DMSO-d_6) δ 8.00 (d, *J* = 8.4 Hz, 4H), 7.77 (d, *J* = 8.4 Hz, 4H), 7.68 (s, 1H), 7.39 – 7.32 (m, 4H), 7.31 (s, 2H), 7.17 – 7.07 (m, 6H).

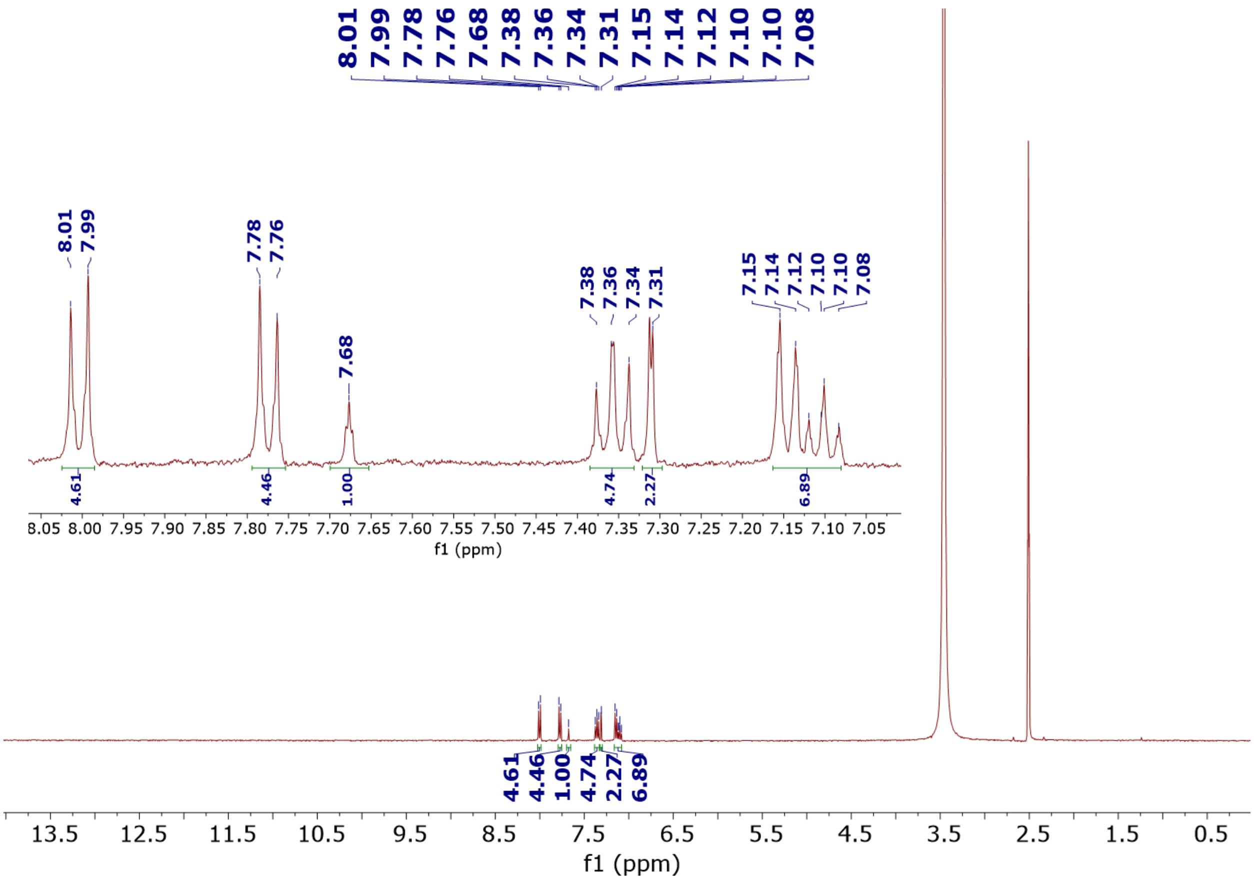

Figure S1. ^{1}H NMR spectrum of primary ligand H_2L_0.

Angular ditopic ligand H_2L_1 was synthesized *via* two coupling reactions followed by saponification in a basic aqueous solution (Scheme S2). Below is the detailed synthesis:

Synthesis of 9-(3,5-dibromophenyl)-9H-carbazole (3): A mixture of 9H-carbazole (4.6 g, 27.5 mmol), sodium hydride (1.2 g, 50.0 mmol) was stirred in dry N, N-dimethylformamide (100 mL) at room temperature under Ar. After 30 min, 3,5-dibromo-fluorobenzene (4.38 g, 25 mmol) was added. Then the mixture was stirred at 155 °C for 12 h. After cooling to room temperature, the reaction mixture was poured into water three times and extracted by CH_2Cl_2, dried over anhydrous $MgSO_4$. After filtration and evaporation, the crude product was purified by column chromatography on silica gel (hexane/dichloromethane = 1:1, v/v), and then concentrated under

reduced pressure to afford a white solid in 56% yield. ^{1}H NMR (400 MHz, Chloroform-*d*) δ 8.15 (dt, *J* = 7.7, 1.0 Hz, 2H), 7.79 (t, *J* = 1.7 Hz, 1H), 7.73 (d, *J* = 1.7 Hz, 2H), 7.48 – 7.42 (m, 4H), 7.35 (ddd, *J* = 8.0, 6.5, 1.7 Hz, 2H).

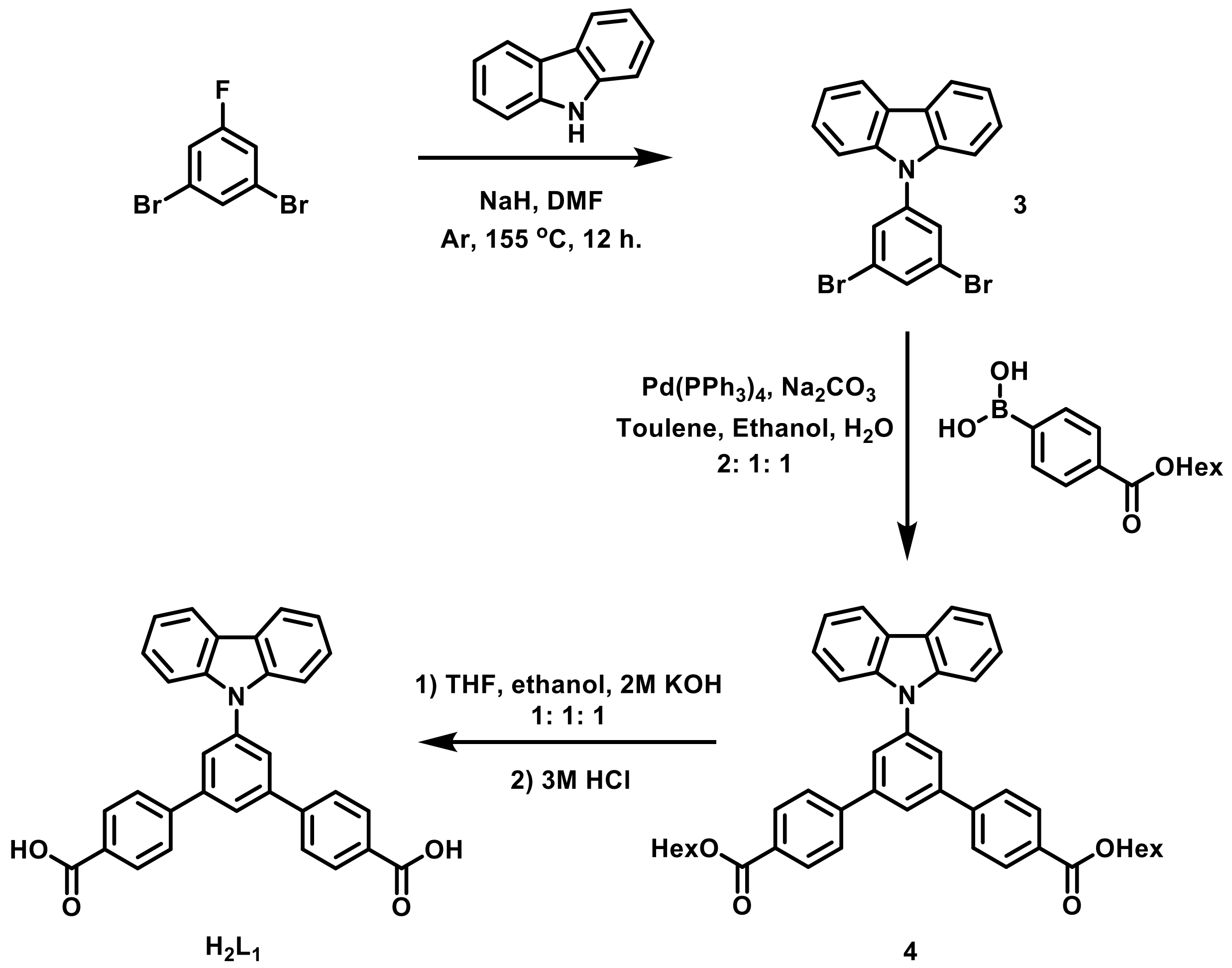

Scheme S2. Synthesis of ligand H_2L_1.

Synthesis of dihexyl 5'-(3,6-di-tert-butyl-9H-carbazol-9-yl)-[1,1':3',1''-terphenyl]-4,4''-dicarboxylate (4): Compound 3 (4.01 g, 10 mmol), (4-((hexanoyloxy)carbonyl) phenyl) boronic acid (6.6 g, 25 mmol), Na_2CO_3 (8.5 g, 80 mmol) were dissolved in mixed solvent of toluene-ethanol-water (40 mL: 20 mL: 20 mL). After degassing by argon for 1 h, Pd $(PPh_3)_4$ (0.8 g, 0.68 mmol) was added to the solution. The solution was stirred under argon atmosphere for 48 h under reflux at 100 °C. After filtration, the solvent was removed under reduced pressure, the

resulting residue was purified using column chromatography of silica gel to obtain compound 4 in 71% yield. ^{1}H NMR (400 MHz, Chloroform-*d*) δ 8.23 – 8.14 (m, 6H), 7.97 (d, *J* = 1.7 Hz, 1H), 7.89 (d, *J* = 1.6 Hz, 2H), 7.80 (d, *J* = 8.3 Hz, 4H), 7.55 (d, *J* = 8.2 Hz, 2H), 7.51 – 7.44 (m, 2H), 7.36 (t, *J* = 7.5 Hz, 2H), 4.38 (t, *J* = 6.6 Hz, 4H), 1.82 (p, *J* = 6.8 Hz, 4H), 1.49 (t, *J* = 7.6 Hz, 4H), 1.39 (dt, *J* = 7.3, 3.7 Hz, 8H), 0.93 (d, *J* = 4.4 Hz, 6H).

Synthesis of 5'-(9H-carbazol-9-yl)-[1,1':3',1''-terphenyl]-4,4''-dicarboxylic acid (H_2L_1): Compound 4 (6.8 g, 10 mmol) was dissolved in a mixture of methanol (60 mL), THF (60 mL) and 2 M KOH (60 mL). The solution was degassed for 1 hour, and then refluxed under argon for 24 h and the solution became clear. Solvent was removed under reduced pressure, and the remaining solid was dissolved in water. 3 M HCl was added to the solution until pH = 2.0. After filtration, precipitate was recrystallized from DMF/water to obtain light yellow solid in 92 % yield. ^{1}H NMR (400 MHz, DMSO-d_6) δ 13.05 (s, 2H), 8.29 (d, *J* = 7.6 Hz, 2H), 8.23 (s, 1H), 8.07 (s, 8H), 8.03 (d, *J* = 1.7 Hz, 2H), 7.57 (d, *J* = 8.3 Hz, 2H), 7.48 (ddd, *J* = 8.3, 7.1, 1.2 Hz, 2H), 7.36 – 7.31 (m, 2H).

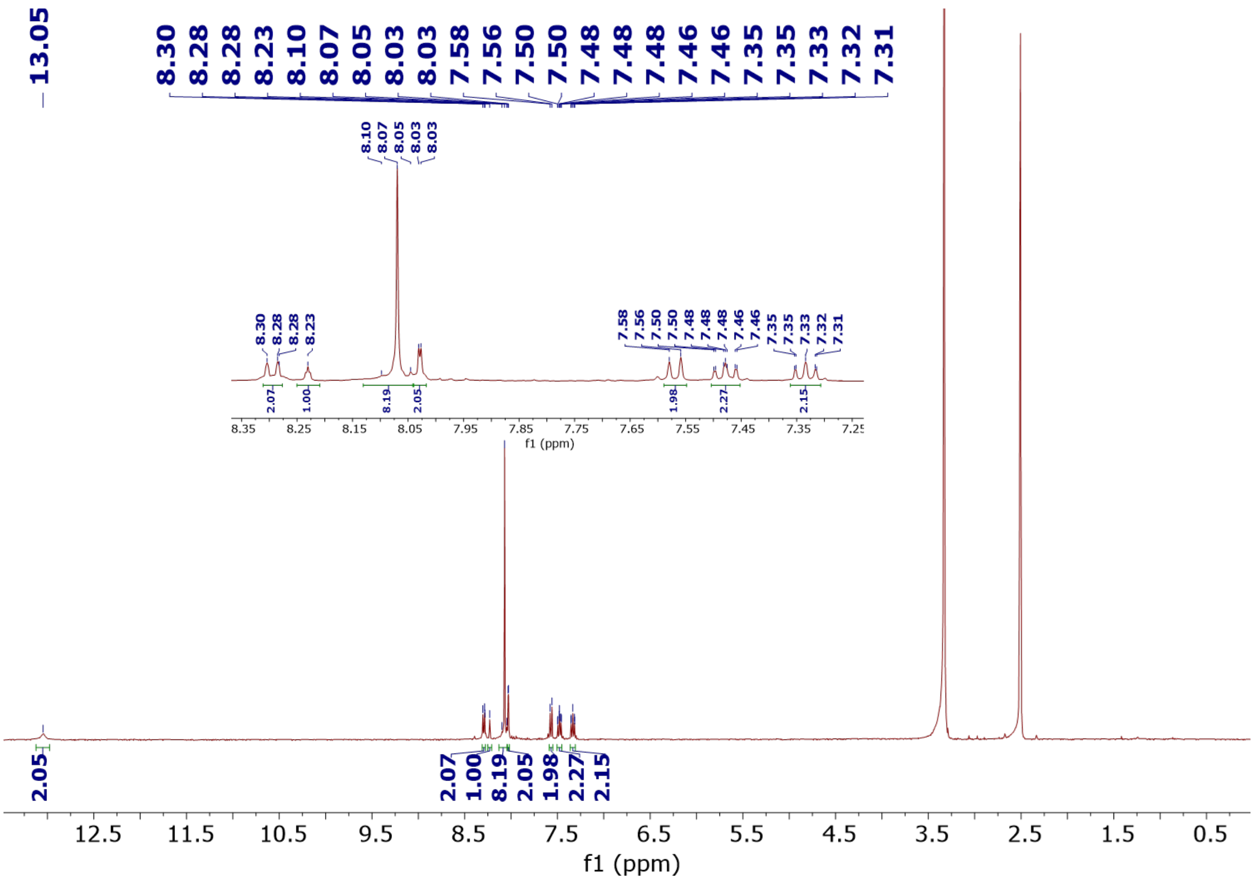

Figure S2. ^{1}H NMR spectrum of primary ligand H_2L_1.

Angular ditopic ligand H_2L_2 was synthesized *via* two coupling reactions followed by saponification in a basic aqueous solution (Scheme S3). Below is the detailed synthesis:

Synthesis of 3,6-dibutylcarbazole: 20 mL CH_2Cl_2 solution containing 2-chloro-2-methylpropane (4.12 g, 30.00 mmol) dropwise added to the 50 mL dry CH_2Cl_2 solution containing carbazole (2.51 g, 15.00 mmol) and $AlCl_3$ (2.00 g, 15.00 mmol) at ice-water bath under Ar, then stirred at room temperature about 12 h. The reaction was quenched by cold water and further extracted by CH_2Cl_2 (30 mL x 3). The organic solvent was removed under reduced pressure. The target compound was finally purified by recrystallization (ethanol) to give the white solid in 75%

yield. ^{1}H NMR (400 MHz, Chloroform-*d*) δ 8.10 (q, *J* = 0.8 Hz, 2H), 7.88 (s, 1H), 7.48 (dd, *J* = 8.5, 1.9 Hz, 2H), 7.36 (dd, *J* = 8.6, 0.7 Hz, 2H), 1.47 (s, 18H).

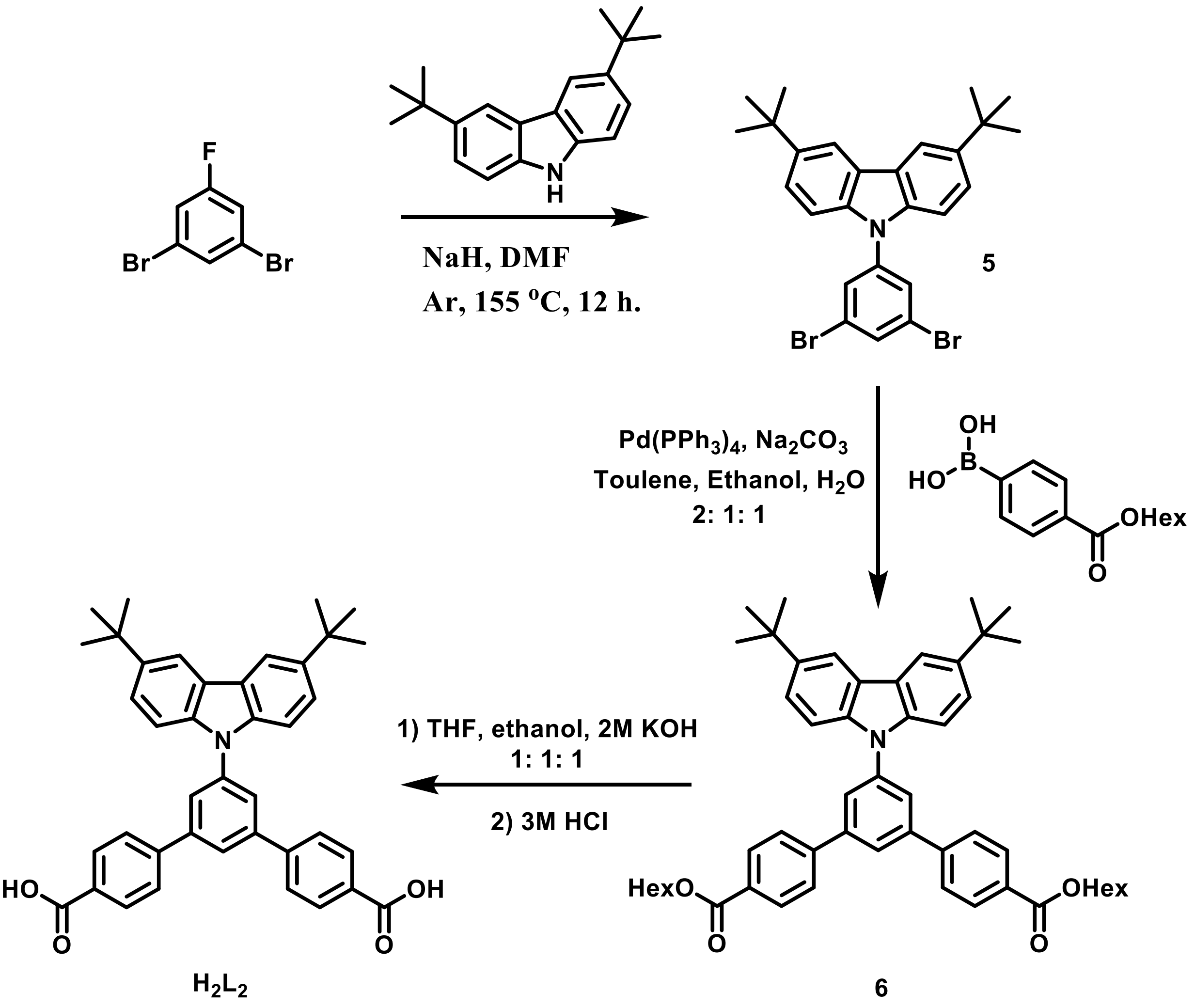

Scheme S3. Synthesis of ligand H_2L_2.

Synthesis of 3,6-di-tert-butyl-9-(3,5-dibromophenyl)-9H-carbazole (5): A mixture of 3,6-dibutylcarbazole (7.67 g, 27.5 mmol), sodium hydride (1.2 g, 50.0 mmol) was stirred in dry N, N-dimethylformamide (100 mL) at room temperature under Ar. After 30 min, 3,5-dibromo-fluorobenzene (4.38 g, 25 mmol) was added. Then the mixture was stirred at 155 °C for 12 h. After cooling to room temperature, the reaction mixture was poured into water three times and extracted by CH_2Cl_2, dried over anhydrous $MgSO_4$. After filtration and evaporation, the crude product was

purified by column chromatography on silica gel (hexane/dichloromethane = 2:1, v/v), and then concentrated under reduced pressure to afford a white solid in 45% yield. ^{1}H NMR (400 MHz, Chloroform-*d*) δ 8.15 (d, *J* = 1.9 Hz, 2H), 7.73 (dd, *J* = 9.0, 1.7 Hz, 3H), 7.52 (dd, *J* = 8.7, 1.9 Hz, 2H), 7.39 (d, *J* = 8.6 Hz, 2H), 1.49 (s, 18H).

Synthesis of dihexyl 5'-(3,6-di-tert-butyl-9H-carbazol-9-yl)-[1,1':3',1''-terphenyl]-4,4''-dicarboxylate (6): Compound 5 (5.13 g, 10 mmol), (4-((hexanoyloxy)carbonyl) phenyl) boronic acid (6.6 g, 25 mmol), Na_2CO_3 (8.5 g, 80 mmol) were dissolved in mixed solvent of toluene-ethanol-water (40 mL: 20 mL: 20 mL). After degassing by argon for 1 h, Pd $(PPh_3)_4$ (0.8 g, 0.68 mmol) was added to the solution. The solution was stirred under argon atmosphere for 48 h under reflux at 100 °C. After filtration, the solvent was removed under reduced pressure, the resulting residue was purified using column chromatography of silica gel to obtain compound 6 in 63% yield. ^{1}H NMR (400 MHz, Chloroform-*d*) δ 8.19 (dd, *J* = 8.2, 1.6 Hz, 6H), 7.93 (t, *J* = 1.7 Hz, 1H), 7.88 (d, *J* = 1.6 Hz, 2H), 7.83 – 7.77 (m, 4H), 7.56 – 7.47 (m, 4H), 4.38 (t, *J* = 6.7 Hz, 4H), 1.85 – 1.79 (m, 4H), 1.51 (s, 22H), 1.39 (dt, *J* = 7.3, 3.7 Hz, 8H), 0.97 – 0.92 (m, 6H).

Synthesis of 5'-(3,6-di-tert-butyl-9H-carbazol-9-yl)-[1,1':3',1''-terphenyl]-4,4''-dicarboxylic acid (H_2L_2): Compound 6 (6.51 g, 10 mmol) was dissolved in a mixture of methanol (60 mL), THF (60 mL) and 2 M KOH (60 mL). The solution was degassed for 1 hour, and then refluxed under argon for 24 h and the solution became clear. The solvent was removed under reduced pressure, and the remaining solid was dissolved in water. 3 M HCl was added to the solution until pH = 2.0. After filtration, the precipitate was recrystallized from DMF/water to obtain a light-yellow solid with a 92 % yield. ^{1}H NMR (400 MHz, DMSO-d_6) δ 13.01 (s, 2H), 8.34 (d, *J* = 1.8 Hz, 2H), 8.18 (t, *J* = 1.7 Hz, 1H), 8.07 (s, 8H), 8.00 (d, *J* = 1.6 Hz, 2H), 7.55 – 7.48 (m, 4H), 1.44 (s, 18H).

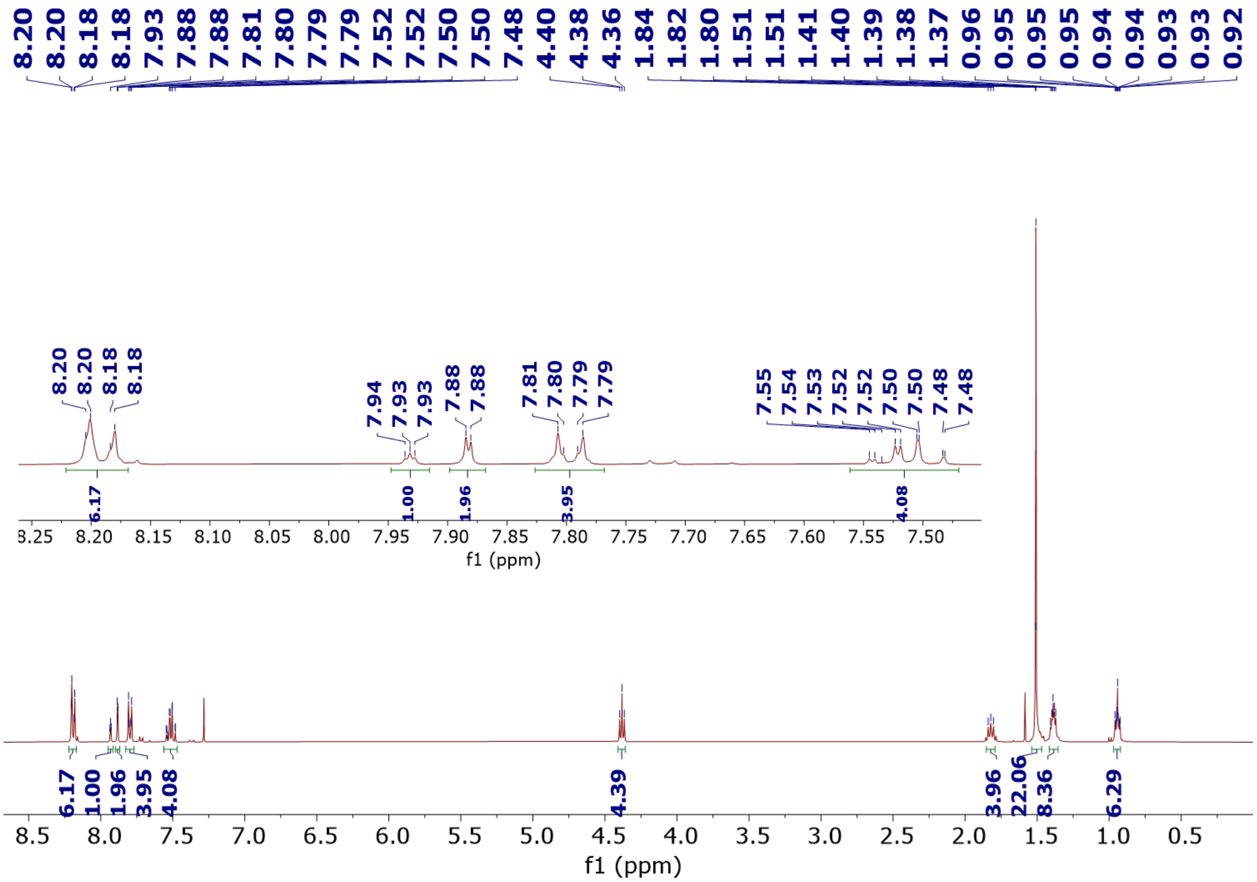

Figure S3. ^{1}H NMR spectrum of compound 6.

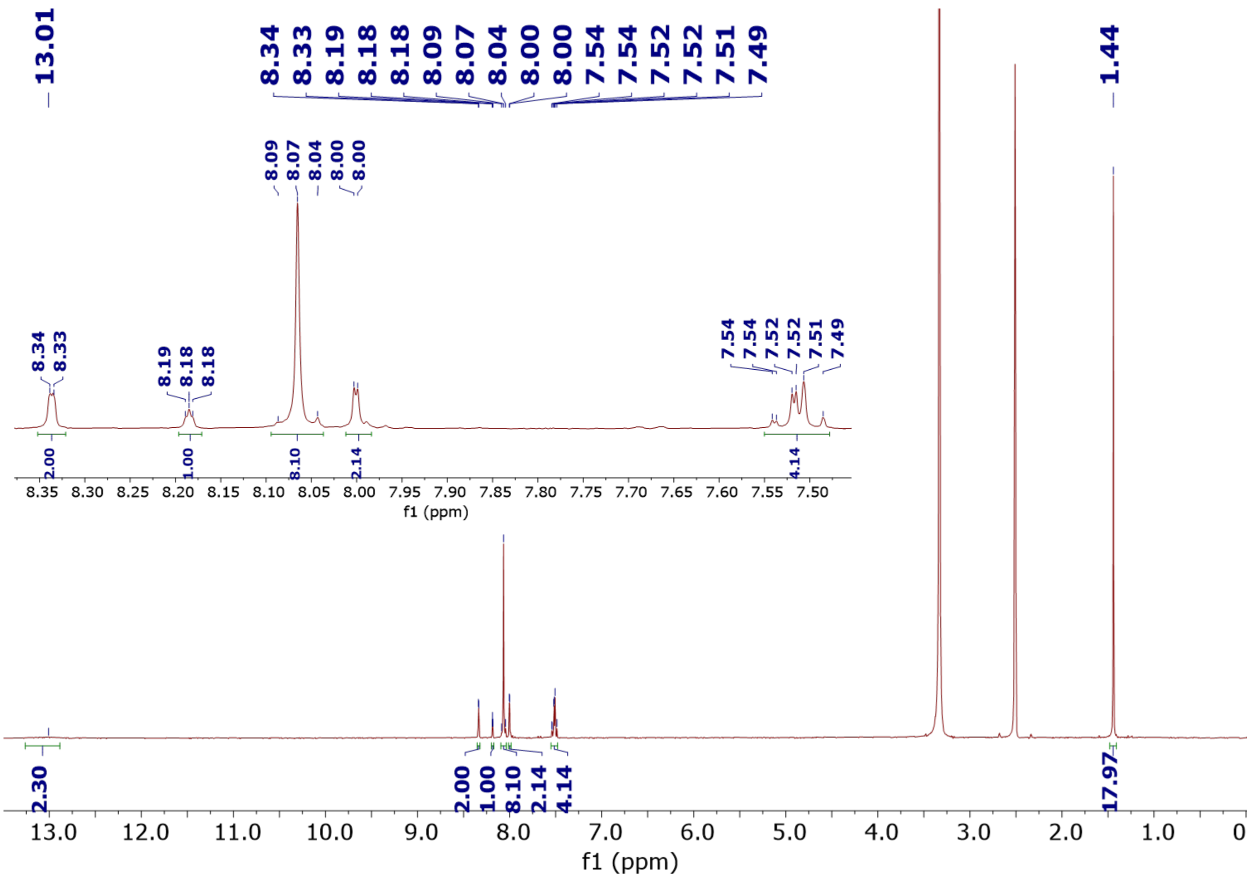

Figure S4. ^{1}H NMR spectrum of primary ligand H_2L_2.

S-3 Synthesis of NPF-800 Series

Synthesis of NPF-800: 2.4 mg of $ZrCl_4 \cdot 8H_2O$ and 20 mg of benzoic acid were mixed in 1 mL DMF in a glass vial and ultrasonically dissolved. The clear solution was heated in an oven at 80 °C for 40 min. After cooling down to room temperature, 7 mg of ligand H_2L_0 and 20 μL trifluoroacetic acid were added to this solution. After capping and sealing the vial, the mixture was sonicated for 5 min to dissolve all ligands. Then the yellow solution was put into an oven and the temperature was increased from 30 °C to 120 °C in 1 h and then kept at 120 °C for 48 h. After cooling down to room temperature for 1 h, colorless octahedron-shaped single crystals were present on the vial wall.

Synthesis of NPF-801: 2.4 mg of $ZrCl_4 \cdot 8H_2O$ and 40 mg of benzoic acid were mixed in 1 mL DMF in a glass vial and ultrasonically dissolved. The clear solution was heated in an oven at 80 °C for 40 min. After cooling down to room temperature, 7 mg of ligand H_2L_1 and 20 μL trifluoroacetic acid were added to this solution. After capping and sealing the vial, the mixture was sonicated for 5 min to dissolve all ligands. Then the yellow solution was put into an oven and the temperature was increased from 30 °C to 120 °C in 1 h and then kept at 120 °C for 48 h. After cooling down to room temperature for 1 h, colorless octahedron-shaped single crystals were present on the vial bottom and wall. As prepared NPF-801 was activated by soaking in fresh DMF at 80 °C for 12 h to remove the unreacted modulators, ligand, and cluster.

Synthesis of NPF-802: 2.4 mg of $ZrCl_4 \cdot 8H_2O$ and 20 mg of benzoic acid were mixed in 1 mL DMF in a glass vial and ultrasonically dissolved. The clear solution was heated in an oven at 80 °C for 40 min. After cooling down to room temperature, 8.5 mg of ligand H_2L_2 and 200 μL acetic acid were added to this solution. After capping and sealing the vial, the mixture was sonicated for 5 min to dissolve all ligands. Then the yellow solution was put into an oven and the

temperature was increased from 30 °C to 120 °C in 1 h and then kept at 120 °C for 48 h. After cooling down to room temperature for 1 h, large colorless octahedron-shaped single crystals were present on the vial bottom and wall. As prepared NPF-802 was activated by soaking in fresh DMF at 80 °C for 12 h to remove the unreacted modulators, ligand, and cluster.

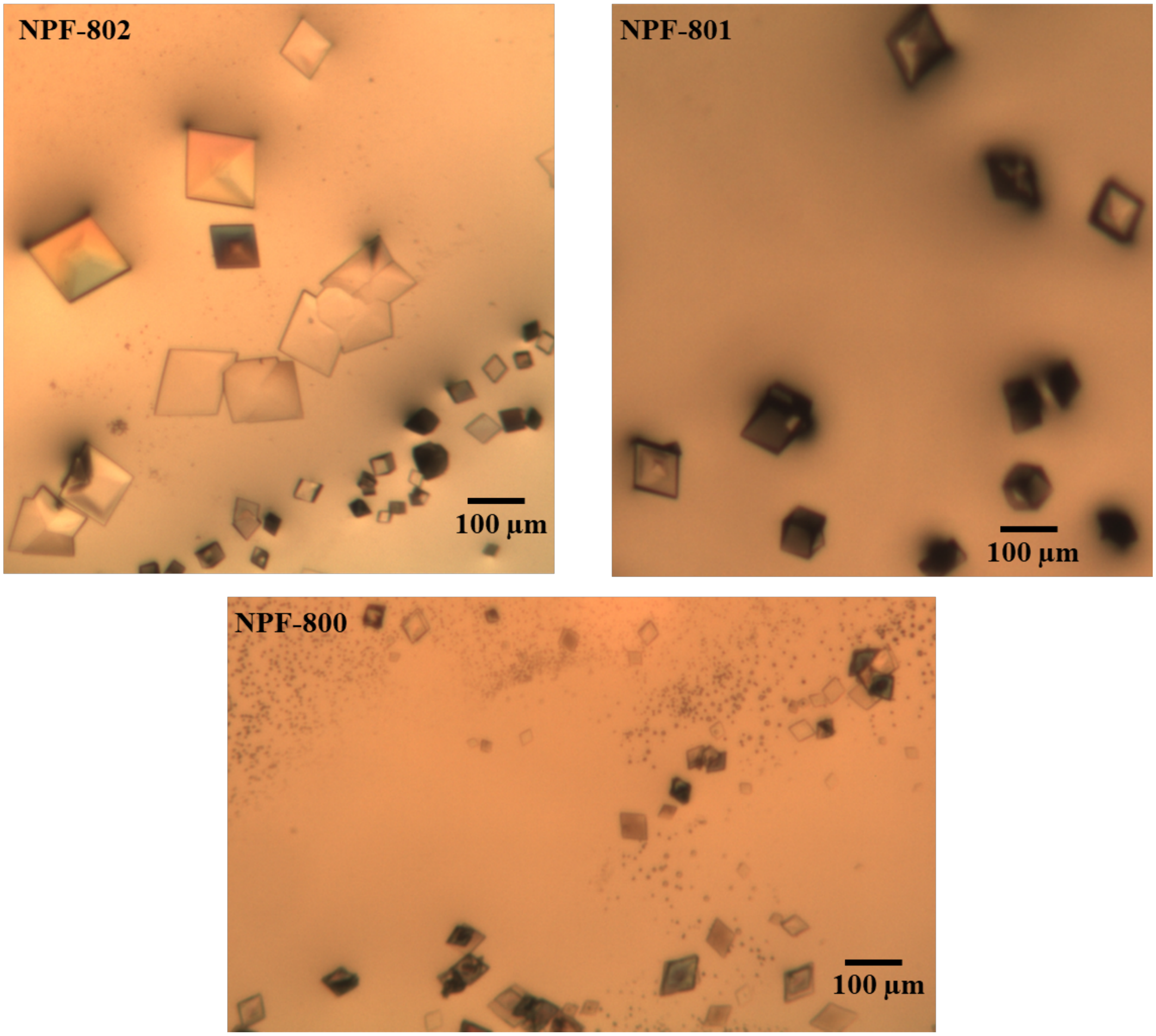

Figure S5. Photos of as-synthesized NPF-800, NPF-801, and NPF-802.

S-4 Powder X-Ray Diffraction

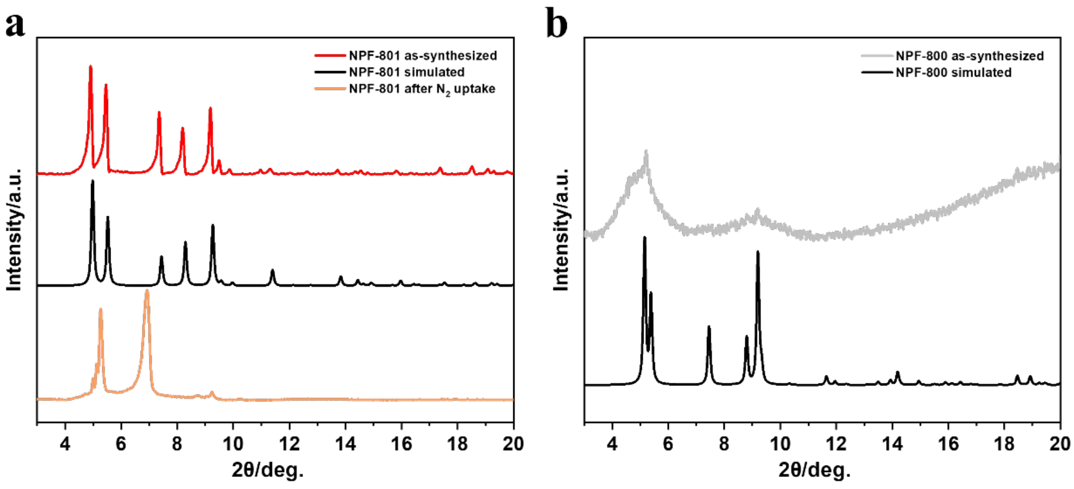

Figure S6. (a) Powder XRD patterns of NPF-801 and NPF-801 after BET N_2 uptake. (b) Simulated powder XRD patterns of NPF-800 and its simulation.

S-5 Crystallographic Data and Structural Representation of NPF-800 Series

All samples were collected from the mother liquid, transferred to oil, then mounted onto glass fiber tips for low temperature (110 K or 120 K) measurement. Crystals were sealed in a glass capillary with mother liquid for room temperature (296 K) measurement. Single crystal X-ray diffraction data was collected using synchrotron radiation, was collected using synchrotron radiation at the Advanced Light Source, Berkeley CA. Indexing was performed using APEX2 (Difference Vectors method). Space groups were determined using XPREP implemented in APEX3. The structure was solved using SHELXS97 (direct methods) and refined using SHELXL-97 within Olex 2 (full-matrix least-squares on F2). Zr, C, O, N atoms were refined with anisotropic displacement parameters and H atoms were placed in geometrically calculated positions and included in the refinement process using the riding model with isotropic thermal parameters: Uiso(H) = 1.2Ueq(-CH). The contributions from disordered solvent molecules were treated as diffusion using the SQUEEZE method implemented in PLATON. Crystal data and refinement conditions are shown in the below tables.

Table S1. Crystal data and structure refinement

Compound name	NPF-802	NPF-801	NPF-800
Empirical formula	$C_{120}H_{105}N_3O_{16}Zr_3$	$C_{96}H_{57}N_3O_{16}Zr_3$	$C_{192}H_{126}N_6O_{32}Zr_6$
Formula weight	2118.72	1782.10	3576.30
Temperature/K	100(2)	273(2)	273(2)
Crystal system	trigonal	trigonal	trigonal
Space group	R-3	R-3	R-3
a/Å	29.7631(18)	31.991(3)	32.8108(9)
b/Å	29.7631(18)	31.991(3)	32.8108(9)
c/Å	28.2604(19)	23.087(4)	21.4661(10)
α/°	90	90	90
β/°	90	90	90
γ/°	120	120	120
Volume/Å^3	21680(3)	20462(5)	20013.2(14)
Z	6	6	3
ρ_{calc}g/cm^3	0.974	0.868	0.890
μ/mm^{-1}	2.135	0.286	0.292
F (000)	6564.0	5412.0	5448.0
Crystal size/mm^3	0.1 × 0.08 × 0.05 mm^3	0.1 × 0.08 × 0.05 mm^3	0.1 × 0.08 × 0.05 mm^3
Radiation	CuKα (λ = 1.54178)	synchrotron (λ = 0.7288)	synchrotron (λ = 0.7288)
2Θ range for data collection/°	5.938 to 133.16	2.354 to 50.842	2.438 to 62.966
Reflections collected	73207	123312	176370
Independent reflections	8520[R_{int}=0.0606 ,R_{sigma}=0.0322]	7812 [Rint = 0.2011, Rsigma = 0.1078]	13647[Rint= 0.1253, Rsigma = 0.0563]
Data/restraints/parameters	8520/298/608	7812/198/332	13647/149/357
Goodness-of-fit on F^2	1.139	1.038	1.052
Final R indexes [I>=2σ (I)]	R1 = 0.1006, wR2 = 0.2783	R1 = 0.1099, wR2 = 0.2810	R1 = 0.0734, wR2 = 0.2420
Final R indexes [all data]	R1 = 0.1251, wR2 = 0.3271	R1 = 0.1422, wR2 = 0.3212	R1 = 0.1035, wR2 = 0.2748
Largest diff. peak/hole / e Å^{-3}	3.45/-1.18	1.69/-1.48	1.12/-0.87

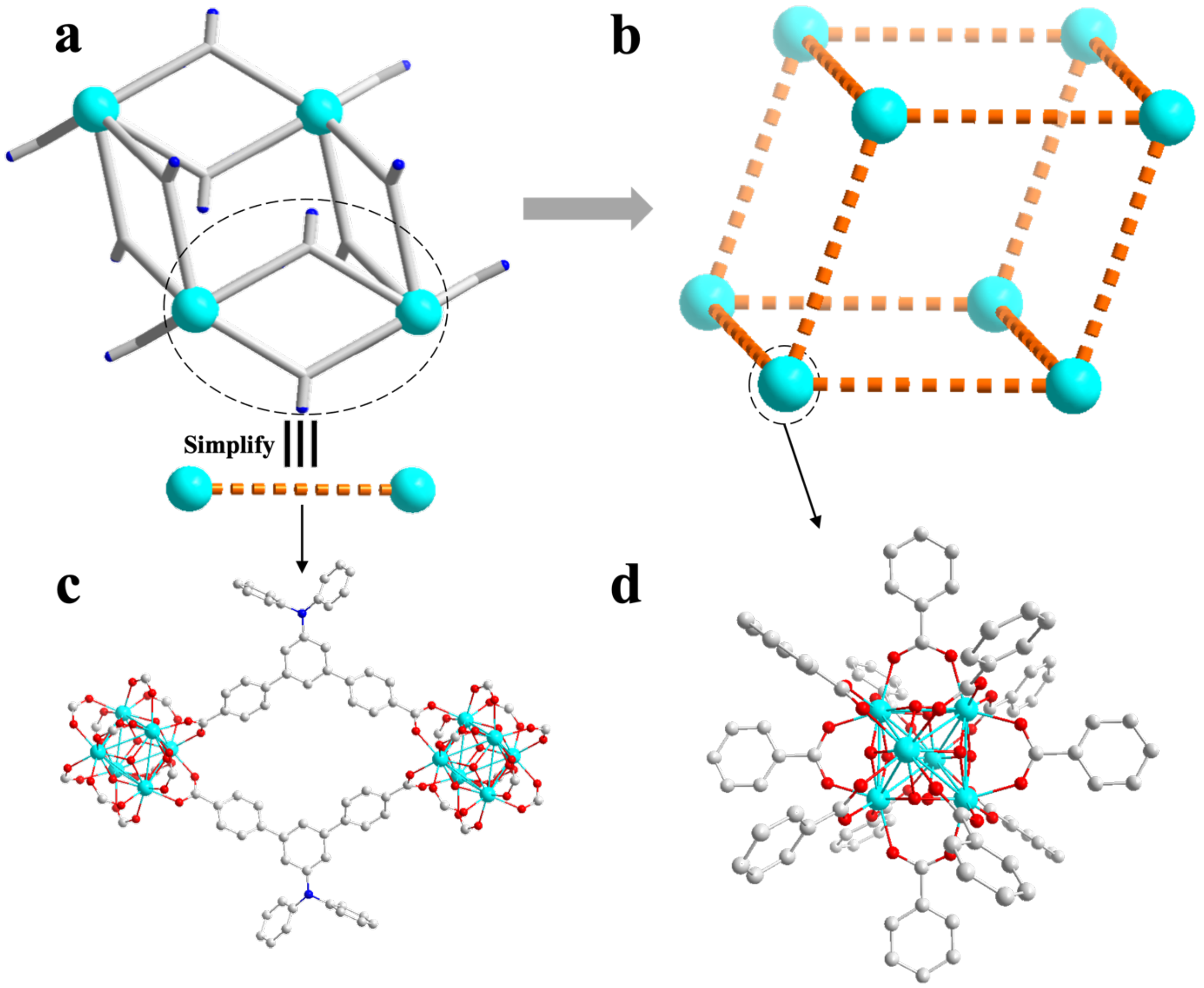

Figure S7. (a) Simplified single network in NPF-800 and (b) its topological representation. (c) The connectivity of primary ligand H_2L_0 in NPF-800. (d) The connectivity of the Zr_6 cluster in NPF-800.

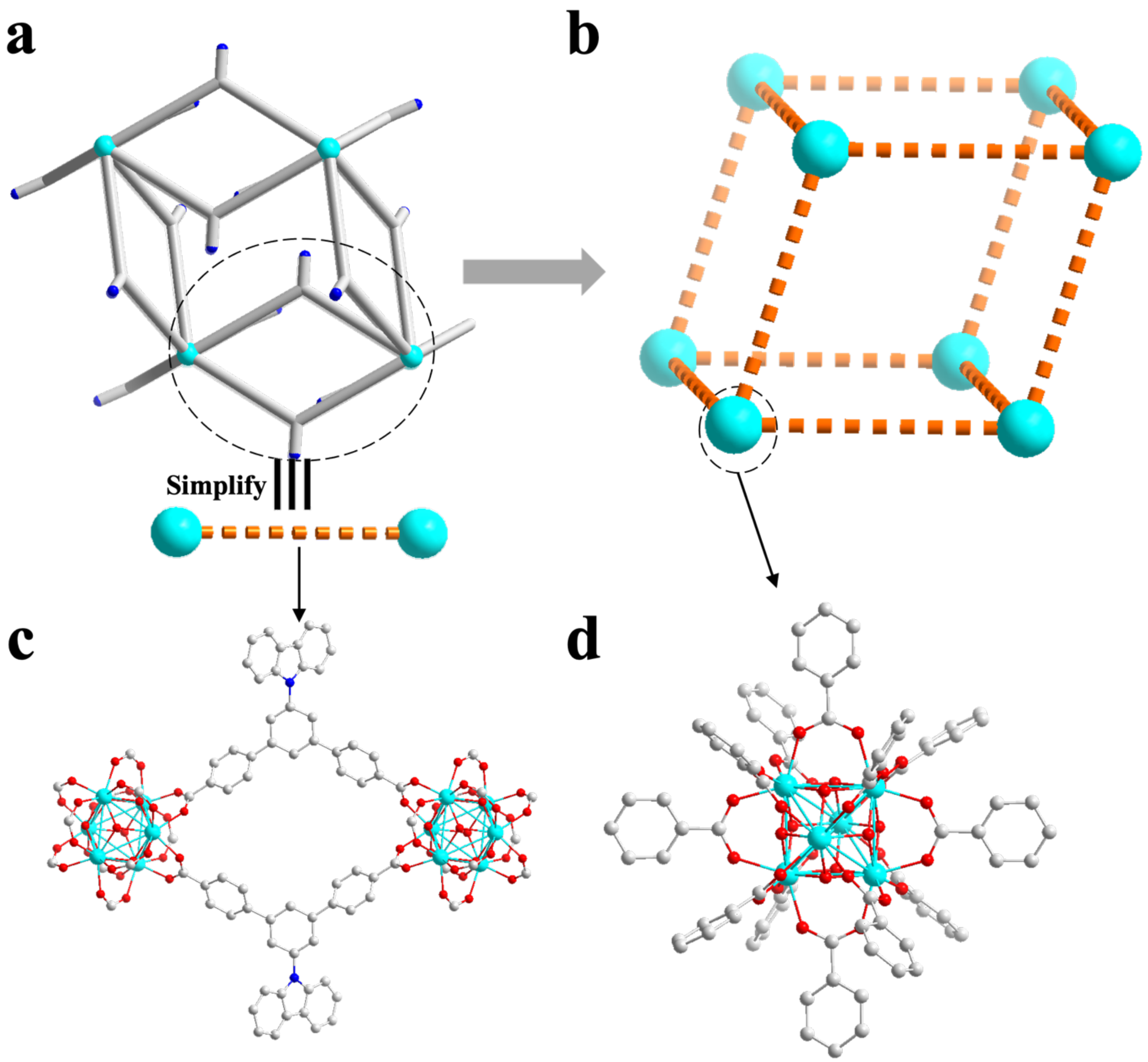

Figure S8. (a) Simplified single network in NPF-801 and (b) its topological representation. (c) The connectivity of primary ligand H_2L_1 in NPF-801. (d) The connectivity of the Zr_6 cluster in NPF-801.

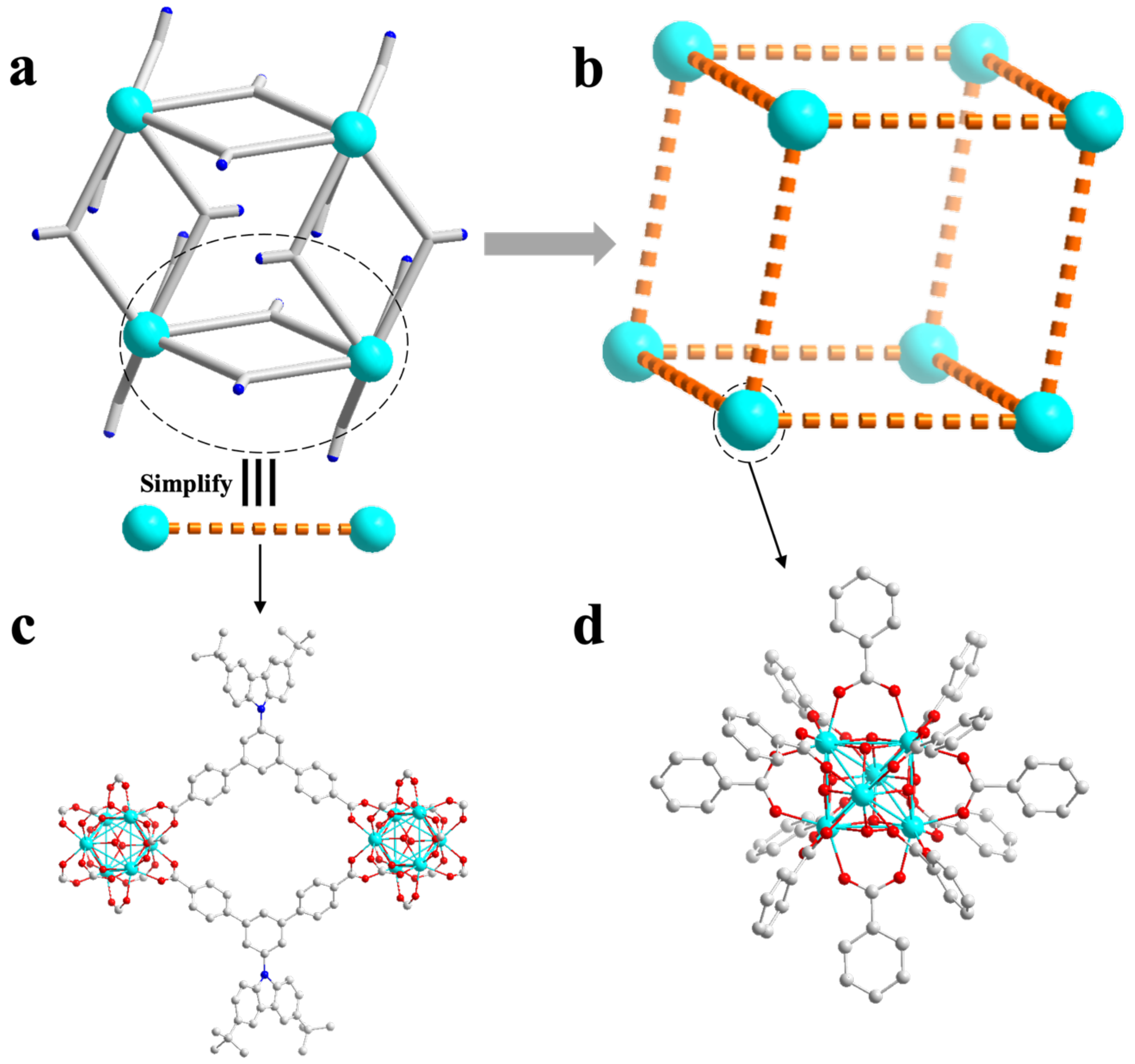

Figure S9. (a) Simplified single network in NPF-802 and (b) its topological representation. (c) The connectivity of primary ligand H_2L_2 in NPF-802. (d) The connectivity of the Zr_6 cluster in NPF-802.

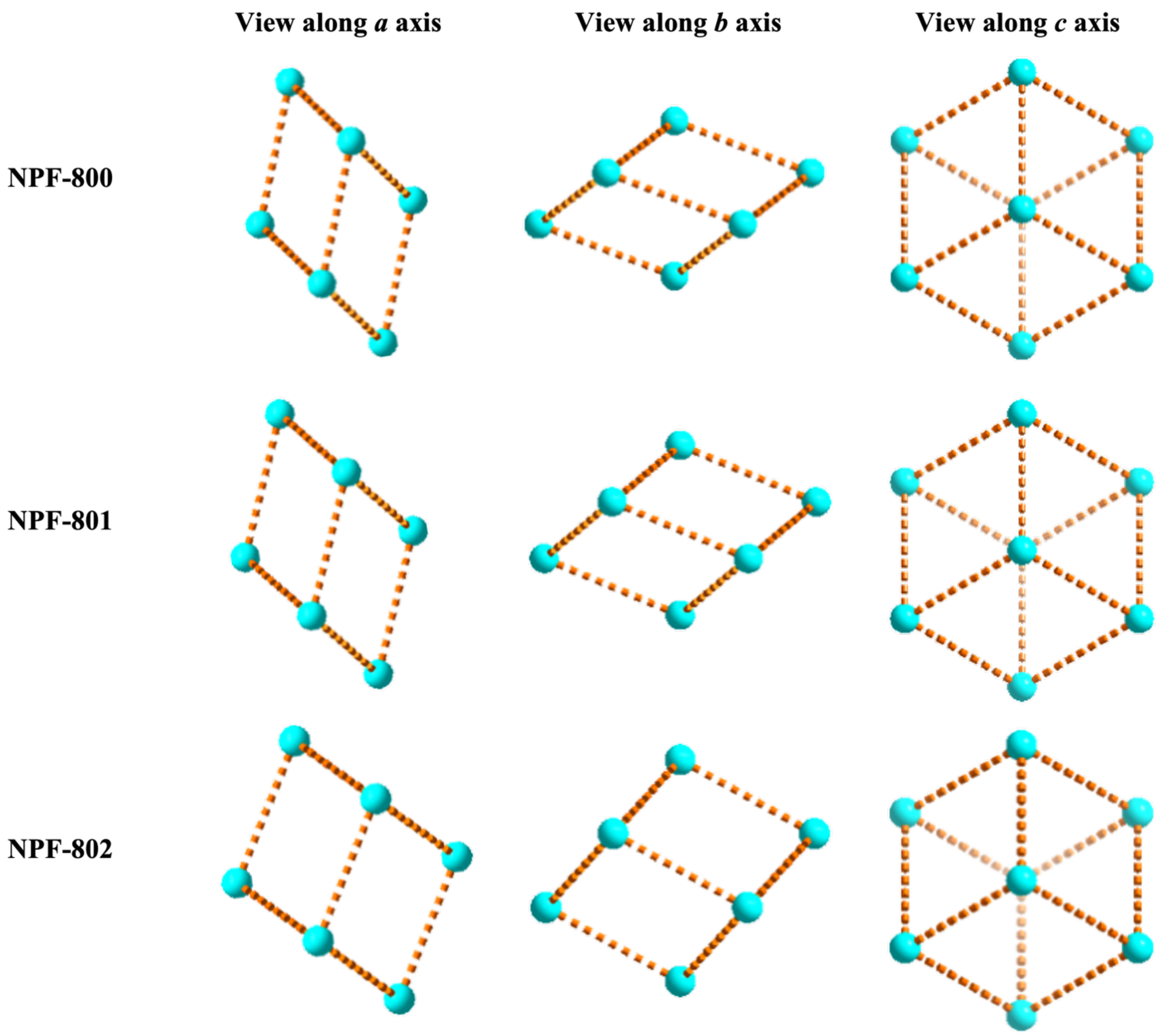

Figure S10. Simplified single network of NPF-800, NPF-801, and NPF-802 view along the *a*, *b,* and *c* axes, respectively.

S-6 Fourier-Transform Infrared Spectroscopy

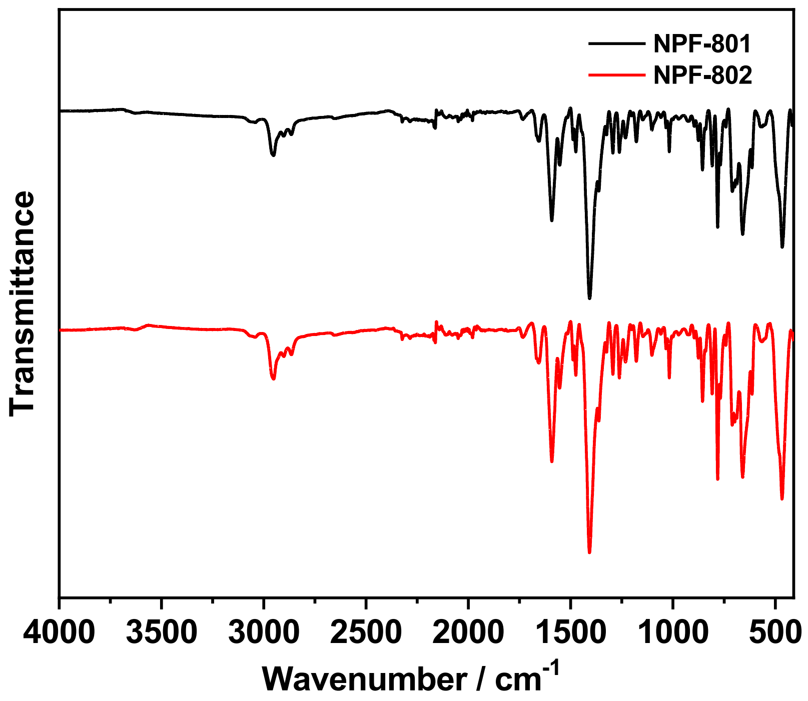

Figure S11. FTIR spectra of NPF-801 and NPF-802.

S-7 Thermogravimetric Analysis

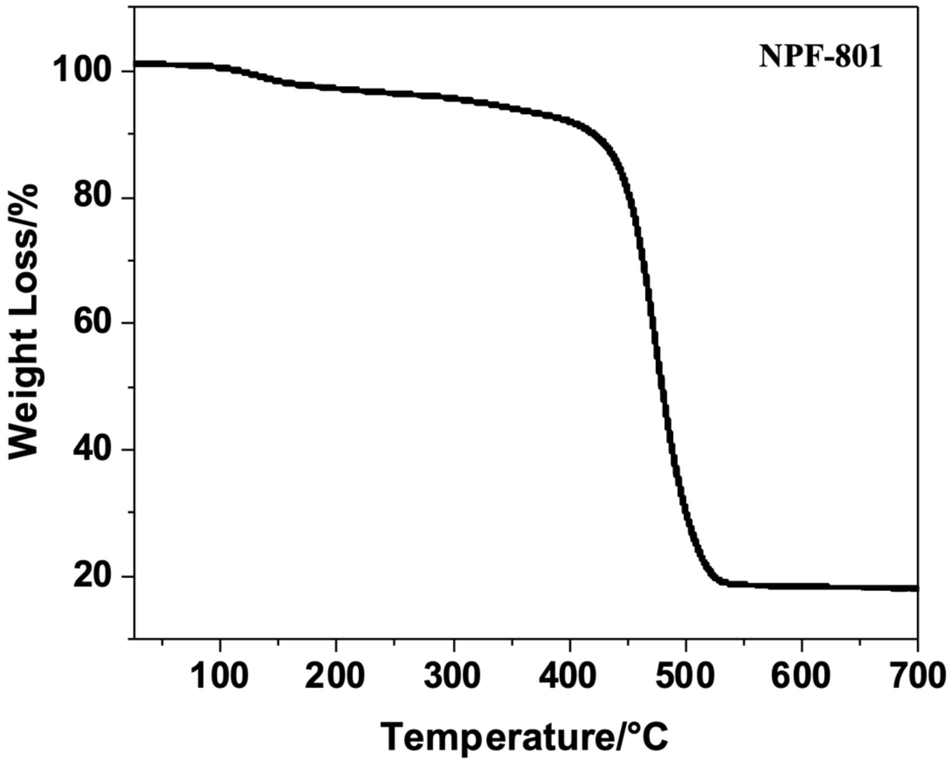

Figure S12. The thermogravimetric analysis (TGA) thermograms of NPF-801.

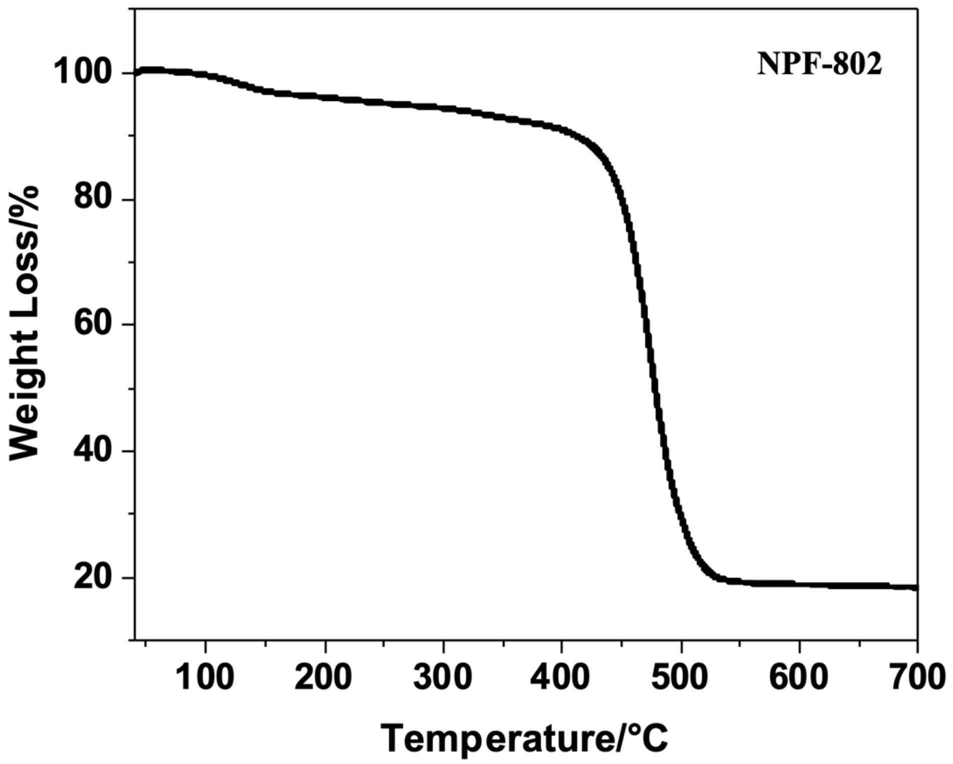

Figure S13. The thermogravimetric analysis (TGA) thermograms of NPF-802.

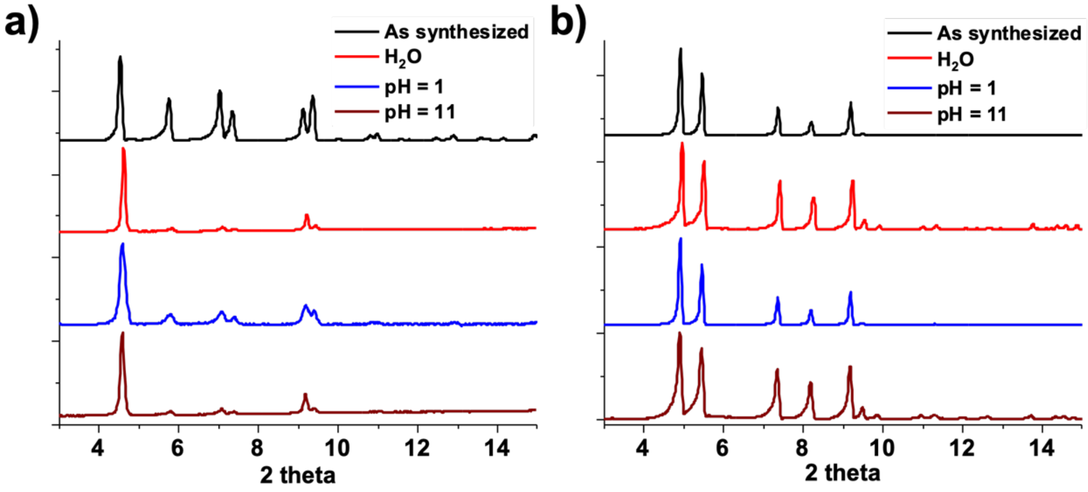

Figure S14. PXRD patterns of (a) NPF-802 and (b) NPF-801 after treatment in treatment in H_2O, basic (pH = 11), and acidic (pH = 1) conditions

S-8 Gas Adsorption and Surface Area

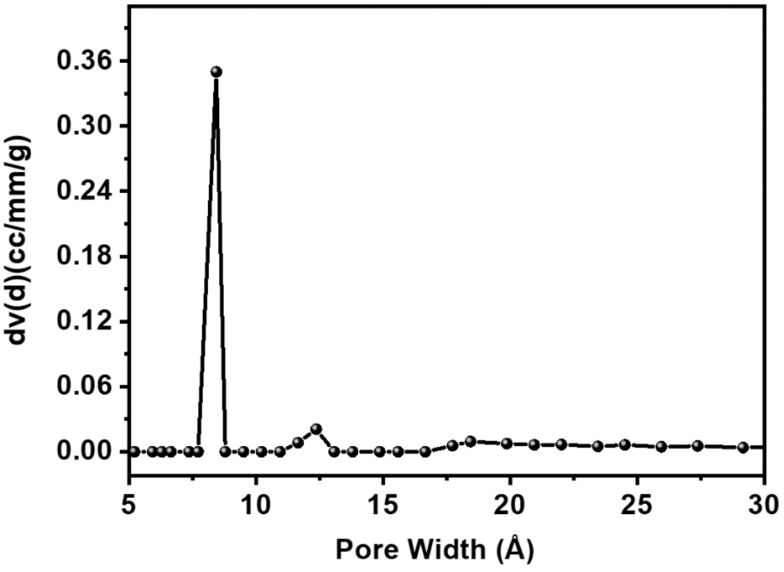

Figure S15. Pore size distribution of NPF-802 based on NLDFT model.

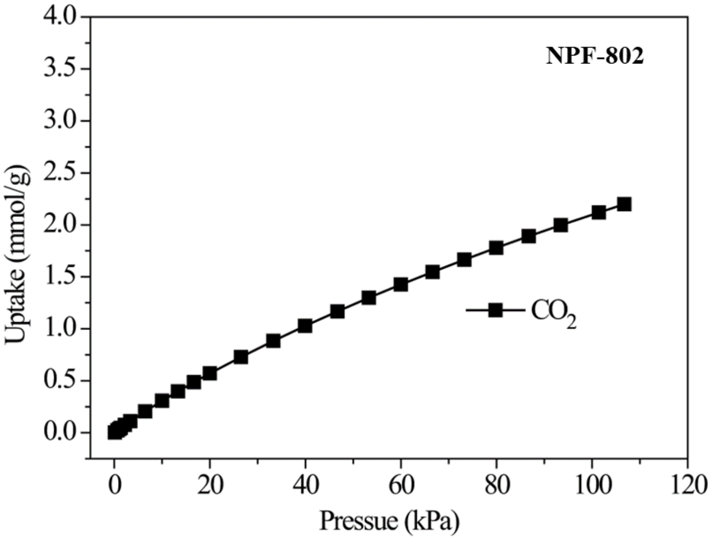

Figure S16. CO_2 adsorption (2.2 mmol g^{-1}) of NPF-802 at 298 K and 1 bar.

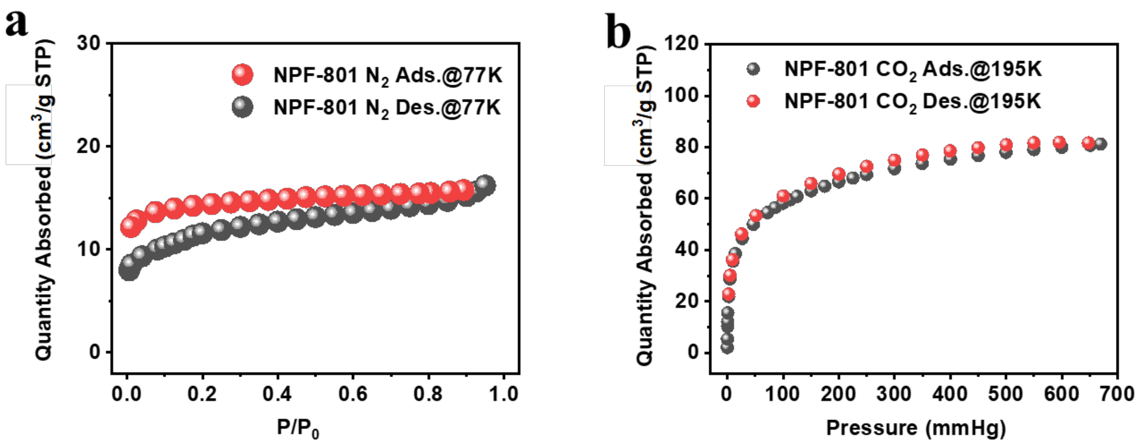

Figure S17. (a) N_2 adsorption isotherms for NPF-801 at 77 K. (b) CO_2 adsorption isotherms for NPF-801 at 195 K.

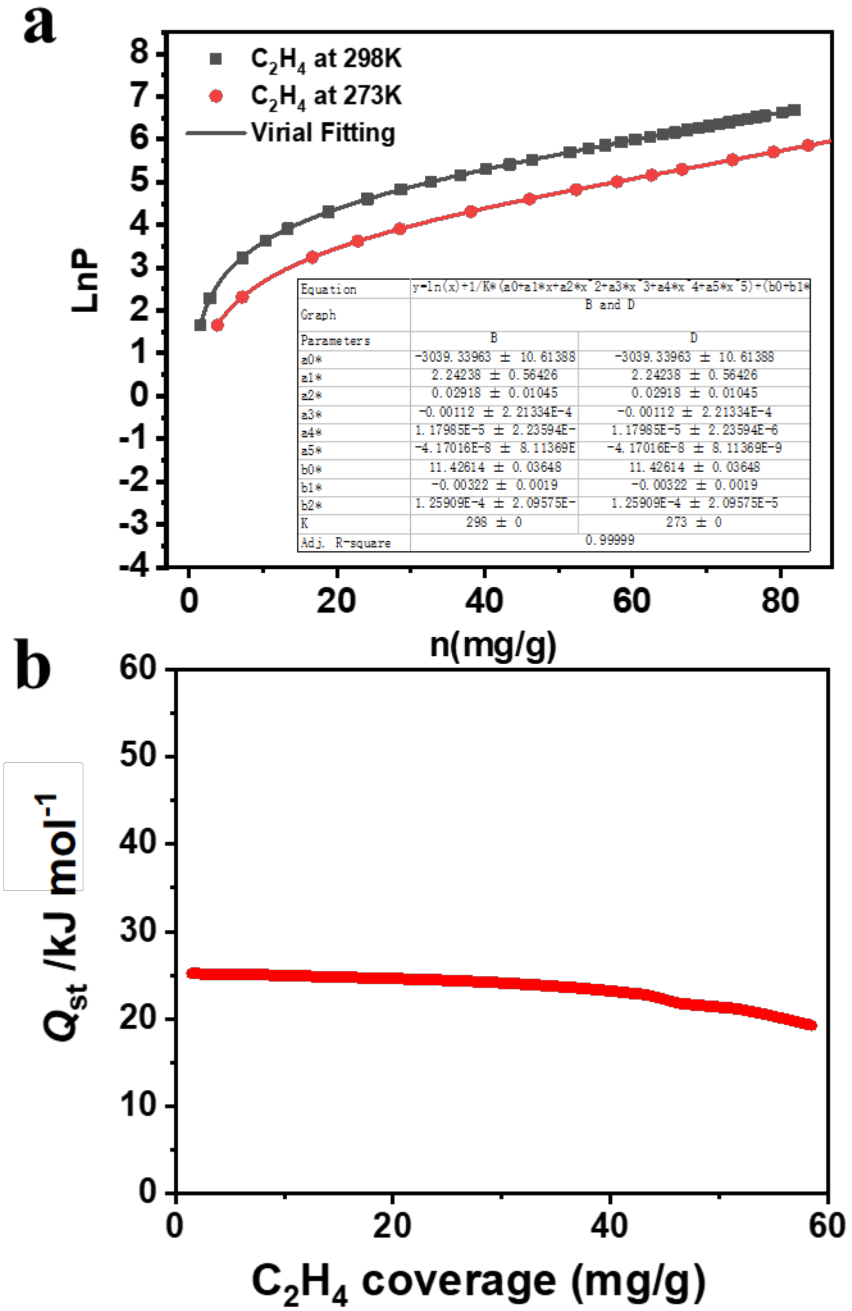

Figure S18. (a) Virial fitting for C_2H_4 isotherm sorption of NPF-802 at 273K and 298K. (b) Coverage-dependent adsorption enthalpy of C_2H_4 of NPF-802 calculated by the virial fitting method.

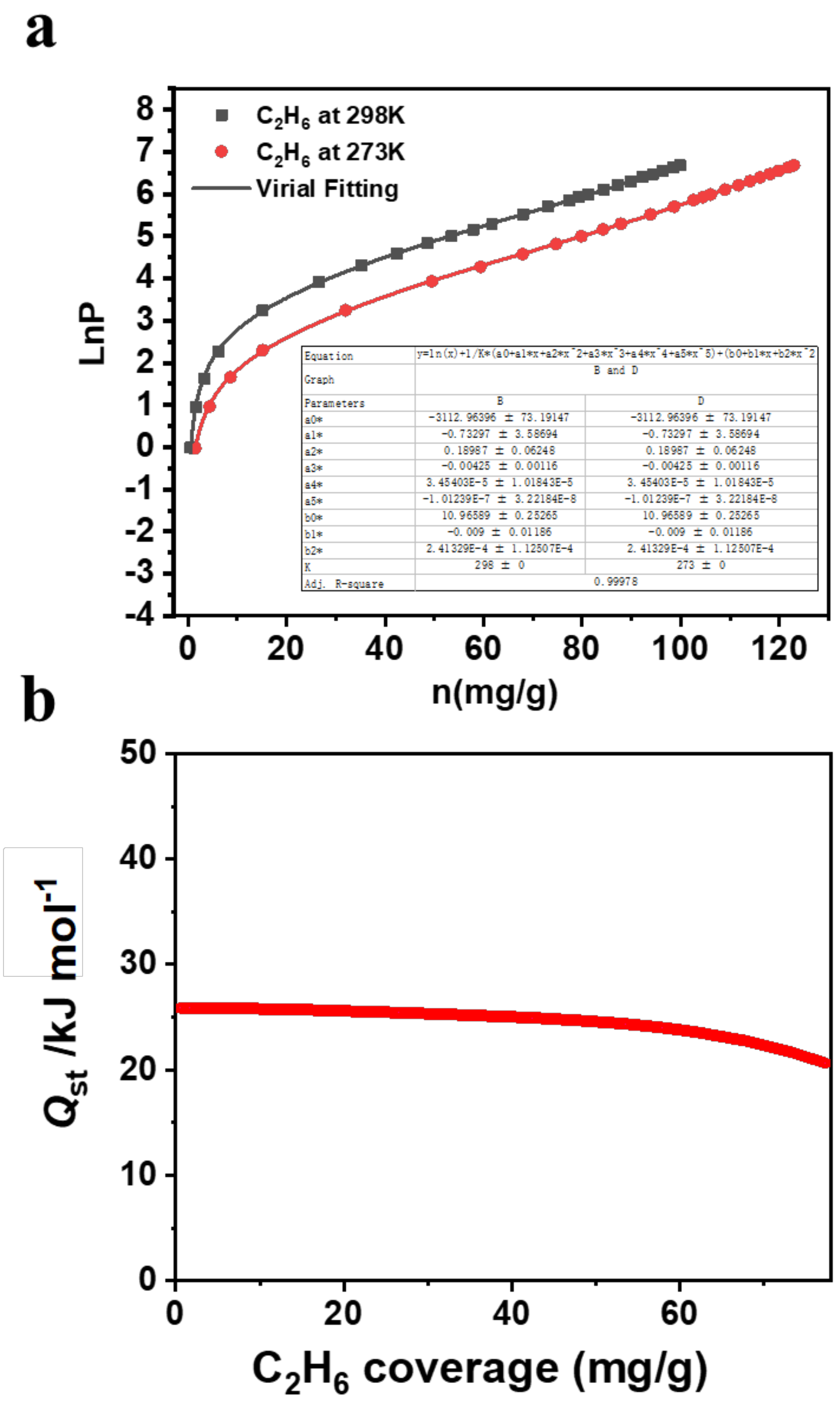

Figure S19. (a) Virial fitting for C_2H_6 isotherm sorption of NPF-802 at 273K and 298K. (b) Coverage-dependent adsorption enthalpy of C_2H_6 of NPF-802 calculated by the virial fitting method.

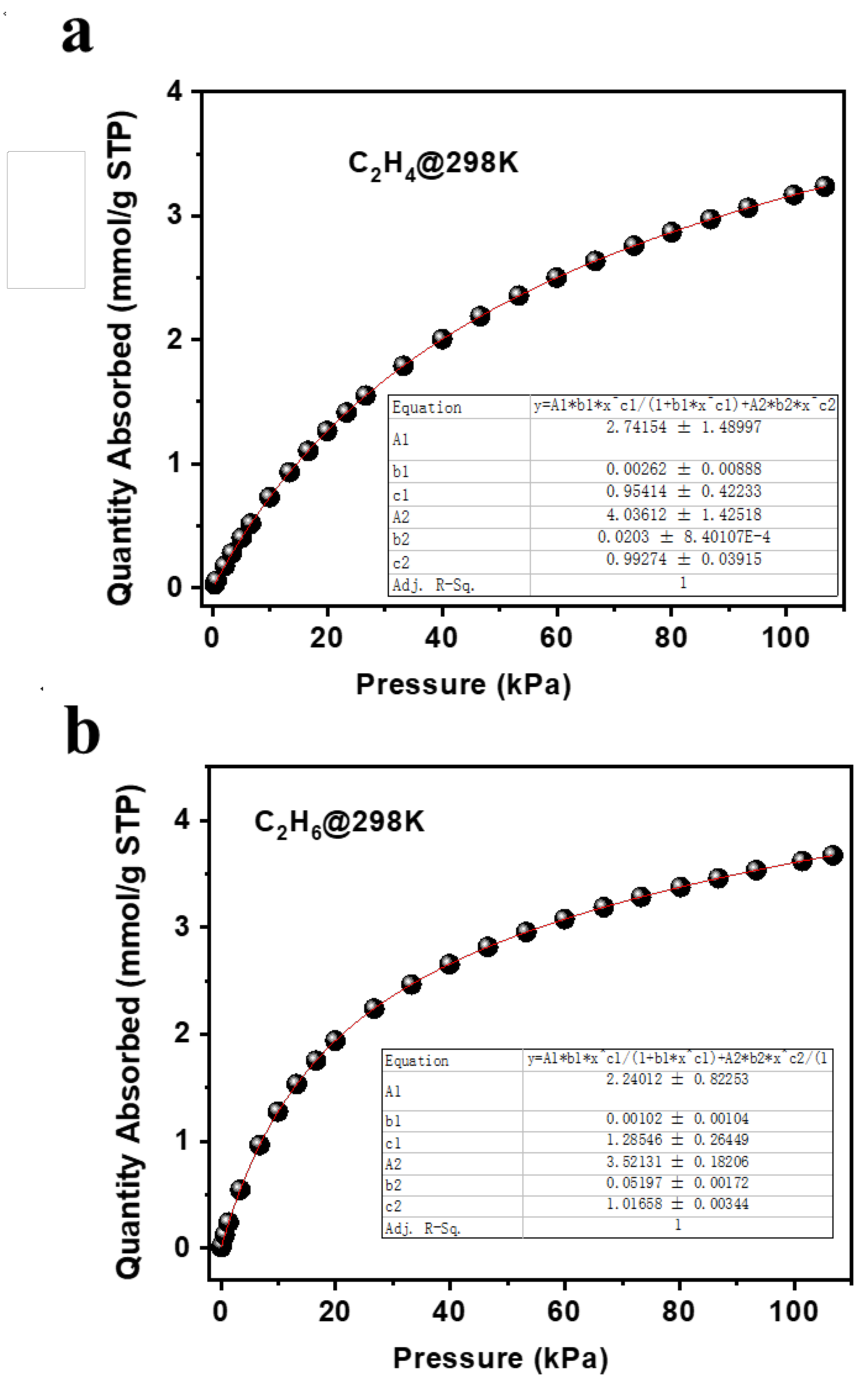

Figure S20. The graphs of the double-site Langmuir-Freundlich equation fitting for C_2H_4 (a) and C_2H_6 (b) isotherms of NPF-802 at 298 K.

Table S2. Summary of the adsorption uptakes (STP), selectivities and heat of adsorption data for various materials.

Materials	C_2H_6 (mmol/g)	(C_2H_4) (mmol/g)	Uptake ratio at 1 bar	IAST Selectivity (50/50)[a]	Q_{st} for C_2H_6 (kJ/mol)[b]	Conditions
NKMOF-8-Br[1]	4.22	3.67	1.15	2.65	40.8	298 K, 1 bar
$Cu(Qc)_2$[2]	1.85	0.78	2.37	3.4	29	298 K, 1 bar
MUF-15[3]	4.69	41.5	1.13	1.96	29.2	298 K, 1 bar
MAF-49[4]	1.72	1.69	1.02	2.7	60	298 K, 1 bar
Mg2V-bdc-tpt[5]	7.45	6.7	1.11	1.6	30.4	298 K, 1 bar
ZIF-8[6]	2.54	1.43	1.78	1.8	17.2	298 K, 1 bar
ZJU-120a[7]	4.91	3.93	1.25	2.74 (296K)	27.6	298 K, 1 bar
$Fe_2(O_2)$(dobdc)[8]	3.4	2.6	1.31	4.4	66.8	298 K, 1 bar
Ni(bdc)$(ted)_{0.5}$[9]	5.0	3.4	1.47	2	21.5	298 K, 1 bar
MIL-142A[10]	3.8	2.9	1.31	1.5	27.3	298 K, 1 bar
PCN-250[11]	5.21	4.22	1.23	1.9	24	298 K, 1 bar
JNU-2[12]	4.1	3.6	1.14	1.6	29.4	298 K, 1 bar
Sc-abtc[13]	4.32	3.99	1.08	1.6	28.2	298 K, 1 bar
UiO-67-$(NH_2)_2$[14]	5.32	4.32	1.23	1.7	26.5	298 K, 1 bar
IRMOF-8[1]	5.02	4.78	1.05	1.8	NA	298 K, 1 bar
NPU-1[15]	4.5	4.2	1.07	1.3	29.1	298 K, 1 bar
**NPF-802**	**3.67**	**3.24**	**1.13**	**1.9**	**25.9**	**298 K, 1 bar**

[a]IAST selectivity, these values are only for the qualitative comparison purpose.

[b]Q_{st} values at low surface coverage.

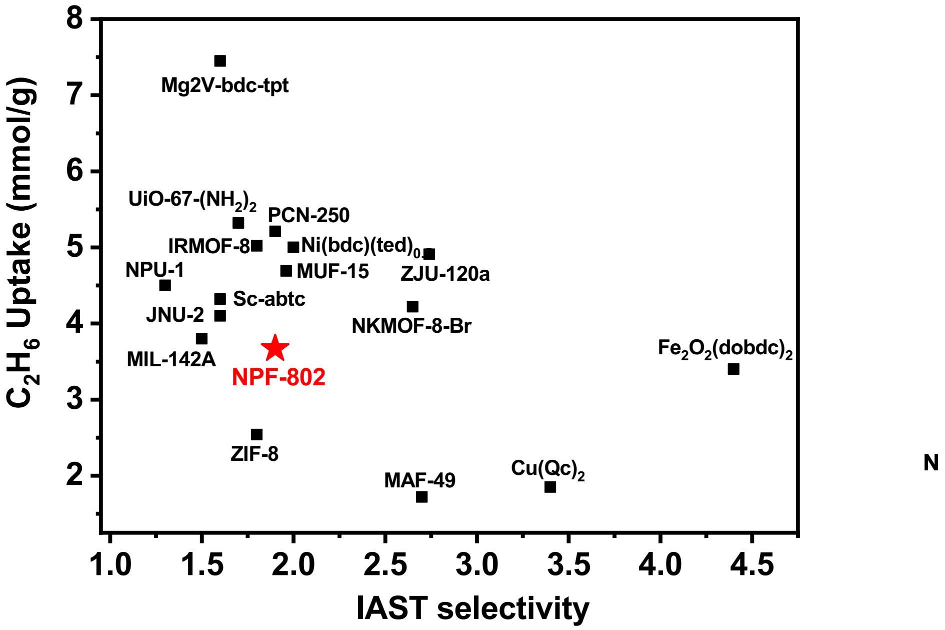

Figure S21. Comparison of IAST selectivities of NPF-802 toward the C_2H_6/C_2H_4 (50:50) mixture and C_2H_6 uptake with previously reported C_2H_6-selective MOFs at 298 K and 1 bar.

References:

1. Geng, S.; Lin, E.; Li, X.; Liu, W.; Wang, T.; Wang, Z.; Sensharma, D.; Darwish, S.; Andaloussi, Y. H.; Pham, T.; Cheng, P.; Zaworotko, M. J.; Chen, Y.; Zhang, Z., Scalable Room-Temperature Synthesis of Highly Robust Ethane-Selective Metal–Organic Frameworks for Efficient Ethylene Purification. *J. Am. Chem. Soc.* **2021,** *143*, 8654-8660.
2. Lin, R.-B.; Wu, H.; Li, L.; Tang, X.-L.; Li, Z.; Gao, J.; Cui, H.; Zhou, W.; Chen, B., Boosting Ethane/Ethylene Separation within Isoreticular Ultramicroporous Metal–Organic Frameworks. *J. Am. Chem. Soc.* **2018,** *140*, 12940-12946.
3. Qazvini, O. T.; Babarao, R.; Shi, Z.-L.; Zhang, Y.-B.; Telfer, S. G., A Robust Ethane-Trapping Metal–Organic Framework with a High Capacity for Ethylene Purification. *J. Am. Chem. Soc.* **2019,** *141*, 5014-5020.
4. Liao, P.-Q.; Zhang, W.-X.; Zhang, J.-P.; Chen, X.-M., Efficient purification of ethene by an ethane-trapping metal-organic framework. *Nature Communications* **2015,** *6*, 8697.
5. Yang, H.; Wang, Y.; Krishna, R.; Jia, X.; Wang, Y.; Hong, A. N.; Dang, C.; Castillo, H. E.; Bu, X.; Feng, P., Pore-Space-Partition-Enabled Exceptional Ethane Uptake and Ethane-Selective Ethane–Ethylene Separation. *J. Am. Chem. Soc.* **2020,** *142*, 2222-2227.
6. Böhme, U.; Barth, B.; Paula, C.; Kuhnt, A.; Schwieger, W.; Mundstock, A.; Caro, J.; Hartmann, M., Ethene/Ethane and Propene/Propane Separation via the Olefin and Paraffin Selective Metal–Organic Framework Adsorbents CPO-27 and ZIF-8. *Langmuir* **2013,** *29*, 8592-8600.
7. Pei, J.; Wang, J.-X.; Shao, K.; Yang, Y.; Cui, Y.; Wu, H.; Zhou, W.; Li, B.; Qian, G., Engineering microporous ethane-trapping metal–organic frameworks for boosting ethane/ethylene separation. *Journal of Materials Chemistry A* **2020,** *8*, 3613-3620.
8. Li, L.; Lin, R.-B.; Krishna, R.; Li, H.; Xiang, S.; Wu, H.; Li, J.; Zhou, W.; Chen, B., Ethane/ethylene separation in a metal-organic framework with iron-peroxo sites. *Science* **2018,** *362*, 443-446.
9. Liang, W.; Xu, F.; Zhou, X.; Xiao, J.; Xia, Q.; Li, Y.; Li, Z., Ethane selective adsorbent Ni(bdc)(ted)0.5 with high uptake and its significance in adsorption separation of ethane and ethylene. *Chem. Eng. Sci.* **2016,** *148*, 275-281.
10. Chen, Y.; Wu, H.; Lv, D.; Shi, R.; Chen, Y.; Xia, Q.; Li, Z., Highly Adsorptive Separation of Ethane/Ethylene by An Ethane-Selective MOF MIL-142A. *Industrial & Engineering Chemistry Research* **2018,** *57*, 4063-4069.
11. Chen, Y.; Qiao, Z.; Wu, H.; Lv, D.; Shi, R.; Xia, Q.; Zhou, J.; Li, Z., An ethane-trapping MOF PCN-250 for highly selective adsorption of ethane over ethylene. *Chem. Eng. Sci.* **2018,** *175*, 110-117.
12. Zeng, H.; Xie, X.-J.; Xie, M.; Huang, Y.-L.; Luo, D.; Wang, T.; Zhao, Y.; Lu, W.; Li, D., Cage-Interconnected Metal–Organic Framework with Tailored Apertures for Efficient C2H6/C2H4 Separation under Humid Conditions. *J. Am. Chem. Soc.* **2019,** *141*, 20390-20396.
13. Shi, Y.; Xie, Y.; Arman, H.; Chen, B., A Scandium-based Microporous Metal-Organic Framework for Ethane-Selective Separation. *Zeitschrift für Anorganische und Allgemeine Chemie* **2022,** *648*, e202200151.
14. Gu, X.-W.; Wang, J.-X.; Wu, E.; Wu, H.; Zhou, W.; Qian, G.; Chen, B.; Li, B., Immobilization of Lewis Basic Sites into a Stable Ethane-Selective MOF Enabling One-Step Separation of Ethylene from a Ternary Mixture. *J. Am. Chem. Soc.* **2022,** *144*, 2614-2623.
15. Zhu, B.; Cao, J.-W.; Mukherjee, S.; Pham, T.; Zhang, T.; Wang, T.; Jiang, X.; Forrest, K. A.; Zaworotko, M. J.; Chen, K.-J., Pore Engineering for One-Step Ethylene Purification from a Three-Component Hydrocarbon Mixture. *J. Am. Chem. Soc.* **2021,** *143*, 1485-1492.
